# Direct conversion of human fibroblasts into therapeutically active vascular wall-typical mesenchymal stem cells

**DOI:** 10.1007/s00018-019-03358-0

**Published:** 2019-11-11

**Authors:** Jennifer Steens, Kristian Unger, Lea Klar, Anika Neureiter, Karolin Wieber, Julia Hess, Heinz G. Jakob, Hannes Klump, Diana Klein

**Affiliations:** 1 Institute for Cell Biology (Cancer Research), University Hospital Essen, Medical Faculty, University of Duisburg-Essen, Virchowstr. 173, Ger-45122 Essen, Germany; 2 Research Unit Radiation Cytogenetics and Clinical Cooperation Group “Personalized Radiotherapy in Head and Neck Cancer, Helmholtz Zentrum München, German Research Center for Environmental Health GmbH, Neuherberg, Germany; 3 Department of Radiation Oncology, University Hospital, LMU Munich, Munich, Germany; 4 Institute for Transfusion Medicine, University Hospital Essen, University of Duisburg-Essen, Essen, Germany; 5 Department of Thoracic and Cardiovascular Surgery, West-German Heart and Vascular Center Essen, University Duisburg-Essen, Essen, Germany

**Keywords:** Vascular wall, Adult stem cells, HOX code, Stem cell therapy, Lineage conversion, Fibroblast, Radioprotection

## Abstract

Cell-based therapies using adult stem cells are promising options for the treatment of a number of diseases including autoimmune and cardiovascular disorders. Among these, vascular wall-derived mesenchymal stem cells (VW-MSCs) might be particularly well suited for the protection and curative treatment of vascular damage because of their tissue-specific action. Here we report a novel method for the direct conversion of human skin fibroblasts towards MSCs using a VW-MSC-specific gene code (*HOXB7*, *HOXC6* and *HOXC8*) that directs cell fate conversion bypassing pluripotency. This direct programming approach using either a self-inactivating (SIN) lentiviral vector expressing the VW-MSC-specific HOX-code or a tetracycline-controlled Tet-On system for doxycycline-inducible gene expressions of *HOXB7*, *HOXC6* and *HOXC8* successfully mediated the generation of VW-typical MSCs with classical MSC characteristics in vitro and in vivo. The induced VW-MSCs (iVW-MSCs) fulfilled all criteria of MSCs as defined by the International Society for Cellular Therapy (ISCT). In terms of multipotency and clonogenicity, which are important specific properties to discriminate MSCs from fibroblasts, iVW-MSCs behaved like primary ex vivo isolated VW-MSCs and shared similar molecular and DNA methylation signatures. With respect to their therapeutic potential, these cells suppressed lymphocyte proliferation in vitro, and protected mice against vascular damage in a mouse model of radiation-induced pneumopathy in vivo, as well as ex vivo cultured human lung tissue. The feasibility to obtain patient-specific VW-MSCs from fibroblasts in large amounts by a direct conversion into induced VW-MSCs could potentially open avenues towards novel, MSC-based therapies.

**Electronic supplementary material:**

The online version of this article (10.1007/s00018-019-03358-0) contains supplementary material, which is available to authorized users.

## Introduction

Tissue stem cells offer a promising therapeutic option for the prevention and treatment of a number of diseases, such as neurological, autoimmune and cardiovascular disorders, and various cancers, e.g., leukemia [1–6]. Herein, the so-called multipotent mesenchymal stem cells (MSCs) were of particular interest. MSCs have been described to home to particular anatomical sites after transplantation and differentiate into specific cell types to locally replace the damaged tissue. However, today it is widely accepted that MSCs preserve existing functional tissue from further destruction or support regenerative processes by paracrine mechanisms such as the synthesis and secretion of protective growth factors and cytokines [7–9]. It has been suggested that MSCs mediate their function through a ‘hit and run’ mechanism, during which MSCs transiently provide a local source of trophic factors in the local environment during temporary localization to the targeted tissue [7, 10, 11]. Furthermore, MSCs exert immune-regulatory activities as they can suppress the T cell response [6, 12, 13]. These unique properties have promoted the wide application of MSCs in clinical trials to treat a wide range of diseases [4, 14–18]. Transplantation of bone marrow-derived MSCs has established itself as a possible strategy for the treatment of graft versus host disease, myocardial infarction, type 1 diabetes mellitus, chronic obstructive pulmonary disease, multiple sclerosis or autoimmune and inflammatory diseases [19–22]. In addition, MSCs have been genetically modified to enable targeted delivery of a variety of therapeutic agents in malignant diseases [5, 23, 24].

In the adult organism, almost all tissues harbor reservoirs of MSCs that contribute to the maintenance of organ integrity by replacing lost cells or by locally secreting cytokines, thereby supporting repair and healing of tissues [25, 26]. Although bone marrow is the most frequently used source for obtaining MSCs, they can also be obtained from umbilical cord blood, placenta, blood, fetal liver, adipose tissue and, also from the wall of adult blood vessels [27–32]. However, the proportion of MSCs contained in primary isolates is rather low. Therefore, alternative, more accessible sources for MSCs are needed. An alternative method for obtaining MSCs is by directed differentiation of embryonic stem (ES-) or induced pluripotent stem cells (iPSCs), in vitro, which allows for the generation of MSCs in large quantities and with comparable properties [33–36]. iPSCs present an unlimited source for a broad range of applications including in vitro disease modeling, drug development, toxicity testing, as well as in vivo cell-replacement therapies [37–39]. Several studies have reported the successful generation of MSCs from human iPSCs [40–42]. However, their clinical use has been hampered by the tumorigenic potential elicited by undifferentiated iPSCs potentially remaining in the differentiated cell population, the lengthy and inefficient differentiation process, and genomic instability due to suboptimal culture conditions. A possible solution to these drawbacks could be to directly program an easily accessible somatic cell type such as fibroblasts towards MSCs.

Different approaches allowing for direct programming of somatic cells can be distinguished: (i) somatic cells can be reprogrammed into iPSCs, (ii) partial reprogramming: a step-wise de-differentiation manner suggesting that re-programming can be controlled and stopped prior to the acquisition of an embryonic-like signature, (iii) direct lineage conversion using transcription factors defining target cell identity, and (iv) chemical-induced conversion [43–45]. The use of the Yamanaka factors (SOX2, OCT4, KLF4, and cMYC) was reported to achieve fibroblasts conversion into induced pluripotent mesenchymal stem cells (iPMSC) when human fibroblasts were transduced with the respective recombinant proteins [46]. However, beside the differentiation into three embryonic germ layer cells in vitro, these iPMSCs formed teratomas in vivo. Recently one conversion method was established that used a defined cocktail of small molecules and growth factors, to achieve an efficient conversion of fibroblasts into induced MSCs with low safety concerns for disease treatments [47]. Of note, no protocols to convert fibroblasts or into MSC by MSC-specific transcription factors have been reported up to now, due to the lack of identified master regulators [46, 47].

We recently reported the in vitro generation of the vascular wall (VW)-typical MSCs from mouse iPSCs, based on a vascular wall MSC-specific gene code [48, 49]. Herein, a lentiviral vector expressing a small set of human vascular wall MSC-specific HOX genes (*HOXB7*, *HOXC6* and *HOXC8*) was used to directly program iPSCs into VW-MSCs which displayed classical MSC characteristics, both in vitro and in vivo [48]. Here we show now that human skin fibroblasts can be directly converted towards vascular wall-typical multipotent stem cells of mesenchymal nature bypassing pluripotency, using the tissue-specific master regulators, namely our previously defined VW-MSC-specific HOX code that encompasses HOXB7, HOXC6 and HOXC8.

## Materials and methods

### HOX gene expression vector and transduction

This study was approved by the local ethics committee of the University Hospital Essen. Skin biopsies were taken from healthy control persons after informed consent. Samples were then obtained and processed in the laboratory anonymously. Respective fibroblasts were cultivated in fibroblast medium (DMEM high glucose, 10% FCS, 50 U/ml Pen/Strep, 1% sodium pyruvate, 1% glutamine, 1% non-essential amino acids and 0.2% β-mercaptoethanol) [50]. Human fibroblasts were transduced using a lentiviral SIN vector co-expressing the coding sequences of *HOXB7*, *HOXC6* and *HOXC8* and the gene encoding *Turquoise2* (cyan) fluorescent protein, all separated by 2A esterase elements or control plasmid (same vector without *HOX* genes) [48]. For this purpose, vector containing supernatants were collected from HEK293 cells transfected with 5 µg of pRRL.PPT.SF.HOXB7.2A.C6L.2A.C8.2A.Turq plasmid or 5 µg of control plasmid, together with 15 µg of a Gag-Pol plasmid and 2 µg of a expression plasmid for VSV-G pseudotyping (pMDG-VSVG). Lentiviral vector particles were concentrated by ultracentrifugation at 27,000×*g*, 1.5 h, 4 °C. Fibroblasts were seeded as single cells at a density of 2 × 10^4^ cells/cm^2^ onto 6-well plates and cultured in fibroblast media with 50 µl of vector-containing supernatants. After 48 h, MSC differentiation was supported by culturing in hMSC-GM media (PromoCell, Heidelberg, Germany). The self-inactivating lentiviral vector for doxycyclin-inducible expression of *HOXB7*, *HOXC6* and *HOXC8* (iHOX, Figure S6) was constructed as follows: a plasmid containing the inducible vector backbone, pRRL.PPT.T11-mCherry.PGK.M2.Pre was cut with AgeI, blunted with Klenow fragment of DNA polymerase I and subsequently cut with BsrGI to release the mCherry-CDS fragment. For the *HOX* co-expression cassette, plasmid pRRL.PPT.SF.HOXB7.2A.C6L.2A.C8.2A.mTurq2.Pre.SIN [48] was cut with BamHI, blunted with Klenow fragment and subsequently cut with BsrGI. The coexpression cassette was then isolated and ligated with the vector backbone to generate pRRL.PPT.T11.HOXB7.2A.C6L.2A.mTurq2.PGK.M2.Pre. Transduced cells were treated with doxycycline (0.2–0.5 µg/ml) 48 h after transduction. Mock-transduced fibroblasts with or without doxycycline-treatment were used as control.

### Trilineage differentiation assay

Differentiation of cultivated MSCs into adipocytes, chondrocytes, and osteocytes was done using ready-to-use differentiation media from Lonza (hMSC Differentiation BulletKit-Adipogenic, PT-3004; -Chondrogenic, PT-3003; -Osteogenic, PT-3002) according to the manufactures instructions. Adipogenic differentiation was verified using Oil red staining, chondrogenic differentiation was verified using collagen type II antibody (Santa Cruz) and immunohistochemitry or Alcian Blue staining solution (1% w/v Alcian Blue in acetic acid, pH 2.5), and osteogenic differentiation was verified using NBT (nitro-blue tetrazolium chloride) and BCIP (5-bromo-4-chloro-3′-indolyphosphate p-toluidine salt) staining (Sigma) for alkaline phosphatase activity.

### Matrigel plug assay

This study was carried out in strict accordance with the recommendations of the Guide for the Care and Use of Laboratory Animals of the German Government. All procedures involving mice were approved by the local institutional Animal Care Committee (Regierungspräsidium Düsseldorf Az84-02.04.2012.A137; Az84-02.04.2012.A285; Az84-02.04.2016.A010). Mice were kept under standard conditions (12 h light and dark cycle, food and water ad libitum) in the Central Animal Facility of the University Hospital Essen. Matrigel plugs were performed and collected as previously described [32]. In brief, mice were anesthetized by injection of intraperitoneal Rompun/Hostaket and the pre-cooled GFR-Matrigel-cell solution (200 µl/injection) containing human AS-M5 endothelial cells as well as control or HOX-transduced fibroblasts was injected subcutaneously. At day 14, mice were killed by isoflurane euthanasia and plugs were removed. Plug samples were fixed with 4% paraformaldehyde (PFA) and subjected for paraffin embedding and sectioning. For mouse xenograft teratomas subcutaneous injection of 1 × 10^6^ cells/ml cells was placed onto both hind limbs of immunodeficient NMRI nu/nu mice (Harlan Laboratories). Mice were monitored daily for teratoma growth.

### RNA isolation, cDNA synthesis and quantitative real-time RT-PCR (qRT-PCR) analysis

For RNA isolation, cells were lysed directly in culture dishes as previously described [49, 51]. RNA was isolated using RNeasy Mini Kit and cDNA synthesis with integrated genomic DNA removal was performed using QuantiTect Reverse Transcription (Qiagen, Hilden, Germany) according to the manufacturer’s instructions. Real-time RT-PCR analysis was carried out using the desoxoligonucleotide primers listed in Table S1. The PCR program consisted of an initial denaturation step at 95 °C for 30 s, annealing at 60 °C for 40 s and extension at 72 °C for 30 s, for a total of 25–30 cycles. Specificity of all PCR reactions was tested by parallel reactions using a no-template control. Real-time PCR analysis was performed using SYBR^®^Green PCR Master Mix (Applied Biosystems, Darmstadt, Germany) and standard conditions. The experiments were performed on ABI PRISM^®^ 7000 sequence detection system (Applied Biosystems). Relative transcript levels of analysed genes were normalized to β-actin mRNA (set as 1).

### Immunohistochemistry and immunofluorescence

Paraffin-embedded tissue sections were hydrated using a descending alcohol series, incubated for 10–20 min in target retrieval solution (DAKO, Glostrup, Denmark) and incubated with blocking solution (2% FCS/PBS). After permeabilisation, sections were incubated with primary antibodies overnight at 4 °C. Antigens were detected with anti-rabbit Alexa488 and anti-mouse Alexa555-conjugated secondary antibodies (1/500). Hoechst 33242 iodide was used for nuclei staining. Cells were cultured on gelatine-coated coverslips and were fixed prior staining using 4% paraformaldehyde for 15 min at room temperature (RT). For staining of nuclear proteins, cells were permeabilized by incubation in 0.1% (v v) Triton X-100 for 5 min at RT. After washing and blocking in PBS with 2% normal goat serum (serum of secondary antibody host species; Cell Signaling Technology), the incubation with the primary antibody was performed for 2–4 h at RT. After washing with PBS, fluorescently labelled secondary antibody was applied for 2 h. Cells were counterstained with Hoechst 33242 and embedded in fluorescent mounting medium (DAKO). Specimens were imaged on a Zeiss Axioserver fluorescence microscope using the Axiovision acquisition software from Zeiss. Antibodies are listed in Table S2.

### Western blot analysis

Whole-cell lysates were generated by scraping cells into ice-cold RIPA-P buffer (150 mmol/L NaCl, 1% NP40, 0.5% sodium-desoxycholate, 0.1% sodium-dodecylsulfate, 50 mmol/L Tris/HCL pH 8, 10 mmol/L NaF, 1 mmol/L Na_3_OV_4_), supplemented with a complete Protease-Inhibitor-Cocktail (Roche) and performing 2–3 freeze–thaw cycles. Protein samples (50–100 µg total protein) were subjected to SDS-PAGE electrophoresis and Western blots were done as previously described using HOXB7, HOXC6, HOXC8, GFP (all 1/200), and Nestin (OriGene, 1/500) or β-Actin (1/5000) antibodies [48, 49].

### Microarray-based gene expression analysis

Isolation and purification of human vascular wall-resident MSCs (from human thoracic internal artery (hITA) specimens) were excised as previously described [32, 49]. Total RNA was isolated from these cells as well as from control and *HOX*-transduced fibroblasts after flow cytometric sorting for Turquoise2 fluorescence (*n* = 4 for each group). Total RNA integrity was assessed using the 2100 Bioanalyzer (Agilent Technologies, Santa Clara, CA, USA) in combination with the RNA 6000 Nano Kit (Agilent Technologies). Global gene expression profiling was performed using SurePrint G3 Human Gene Expression 8x60 k microarrays (AMADID 028005, Agilent Technologies) according to the manufacturer’s protocol with an input of 50 ng of total RNA (one-color Low Input Quick Amp Labeling Kit, Agilent Technologies) and as previously described [48]. Data quality assessment, preprocessing, normalization, and differential expression analyses were conducted using the R Bioconductor packages limma [52] and Agi4x44PreProcess, whereas Benjamini–Hochberg adjusted *P* values smaller than 0.05 were considered statistically significant [53]. Unsupervised hierarchical clustering and visualization were performed on gene expression *z* scores using the heatmap.2 function from the R package gplots (https://cran.r-project.org/web/packages/gplots/index.html) with standard options (Eucledian clustering distance and clustering function “complete”). Gene Set Enrichment Analysis (GSEA) using gene sets from the Molecular Signatures Database (GenePattern, MsigDB v.3.0) was used to search for multigene signatures allowing distinguishing classes [54, 55]. Unsupervised hierarchical clustering and visualization were performed on gene expression *z* scores using the heatmap.2 function from the R package gplots with standard options (Eucledian clustering distance and clustering function “complete”). The accession number for the microarray data reported in this article is ArrayExpress: E-MTAB-6743. GSEA (GenePattern, MsigDB) was used to search for multigene signatures allowing distinguishing classes.

### Global DNA methylation analysis

Processing of DNA Methylation arrays was performed on an Illumina (San Diego, CA, USA) platform at the Genome Analysis Center (GAC) of Helmholtz Zentrum München, described as follows. Total gDNA was isolated from primary fibroblasts, control-transduced fibroblasts, generated HOX-transduced VW-MSCs and hITA-derived VW-MSCs (*n* = 4 for each group). Bisulfite conversion of 500 ng of DNA was done using the EZ DNA Methylation Kit (Zymo Research, Orange, CA, USA) as described recently [56]. Converted gDNA was processed using Infinium^®^MethylationEPIC BeadChips (Illumina, San Diego, CA, USA) following the Illumina Infinium HD Methylation instructions as described [56]. GenomeStudio (version 2011.1) with Methylation Module (version 1.9.0) was used to process the raw image data generated by the BeadArray Reader. Initial quality control of assay performance was undertaken using “Control Dashboard” provided by GenomeStudio Software, including the assessment of staining, extension, hybridization, target removal, bisulfite conversion, specificity, negative, and non-polymorphic control and checking for number of detected CpG sites. As human fibroblasts (donor material) were obtained anonymously, the R tutorial for the epigenetic age prediction tool *MethylAger*, integrated into the *RnBeads* software package was used to ensure similar age distributions (Supplementary Table S3) [57–59].

### Cell proliferation and mixed lymphocyte reaction

Cells were fixed with methanol for 10 min at the indicated time points and subsequently stained with 0.5% (w/v) crystal violet dye (suspension in methanol: deionized water, 1:5) for 10 min. Excess crystal violet dye was removed by five washes with deionized water on a shaker (10 min for each washing step) and the culture plates dried overnight. Crystal violet was released from cells by incubation with 1% sodium dodecyl sulfate (SDS) for 1–2 h before optical density measurement at 595 nm. The cell proliferation reagent WST-1 was used as a ready-to-use colorimetric assay for the nonradioactive quantification of cellular, viability and cytotoxicity according to the manufactures instructions. Optical density measurements were performed 60–90 min after incubation at 450 nm. For mixed lymphocyte reactions, HOX- and control-transduced cells as well as VW-MSCs were plated in 96 well plates (5000 cells per well). After adherence, plates were left untreated or irradiated with 10 Gy. After additional 24 h, medium was exchanged and lymphocytes of different lines were added (10,000 cells per well): human lymphoma cells [MOLT17 (ACC 36) T cell leukemia, DoHH2 non-Hodgkin’s B cell lymphoma cells (both from Leibniz Institute DSMZ-German Collection of Microorganisms and Cell Cultures, Braunschweig, Germany), Jurkat, Clone E6-1 peripheral blood T lymphocyte, U937 myeloid lineagee histiocytic lymphoma (both ATCC/LGC Standards). After additional 24 h of co-culture, cell proliferation was determined using the WST-1 colorimetric assay. Values were compared to that one obtained from single lymphocyte cultures.

### Whole thorax irradiation (WTI)

Wildtype C57BL/6 mice (mixed gender) received 15 Gy of WTI in a single dose, as previously described [51]. All procedures involving mice were approved by the local institutional Animal Care Committee (Regierungspräsidium Düsseldorf Az84-02.04.2012.A137; 84-02.04.2012.A034). Single-cell suspensions of cultured (control and HOX-transduced) fibroblasts (0.2 × 10^6^ cells) were intravenously transplanted into the tail vein of WTI mice 24 h after irradiation or in sham irradiated (0 Gy) control animals, as previously described [51, 60]. Mice were sacrificed 25–30 weeks post-irradiation and lung tissues collected for further analysis. Total white blood cell counts as well as differential blood cell parameters (lymphocytes, monocytes, and granulocytes) were analyzed in a scil Vet ABC hematology analyzer (scil animal care company, Gurnee, IL) using 10 µl peripheral blood samples (EDTA anticoagulated) at 3 weeks (within the acute/pneumonitic phase) and 25 weeks (within the chronic/fibrotic phase) after irradiation.

### Lung histopathology

For lung histology, mice were narcotized using isoflurane and killed by transcardial perfusion with PBS, as previously described [51, 60]. In brief, whole inflation fixed lungs were embedded in paraffin. 3 μm longitudinal cross-sections were taken per mouse lung at the midpoint through the lung block depth. Sections were stained with Masson’s Goldner Trichrome (Carl Roth, Karlsruhe, Germany) for histological evaluation. Sections were scored blinded to the genotype and treatment group using a 0–8 point Ashcroft scale [60, 61]. The mean scores (five per section) were averaged to yield the final score for each specimen. Depicted data represent the mean values of all mice per group as indicated.

### Ex vivo lung tissue culture

Normal human lung tissue samples were obtained during surgery according to local ethical and biohazard regulations and provided from the Department of Thoracic Surgery and Surgical Endoscopy, Ruhrlandklinik, University Hospital Essen. Experiments were approved by the local ethics committee (17-7454-BO; Ethikkommission of the University Medical Faculty, Essen, Germany). Cell cultures of human microvascular endothelial cells (AS-M5) and of human lung tissue specimen were performed as previously described [60, 62]. In brief, fresh lung specimens were dissected and embedded in matrigel using 48-well tissue culture plates and incubated at 37 °C in a humidified atmosphere with 5% CO_2_. Embedded pieces as well as cultured endothelial cells were irradiated with 15 Gy using the Isovolt-320-X-ray machine (Seifert-Pantak, East Haven, CT) at 320 kV, 10 mA with a 1.65-mm aluminum filter and further incubated in normal growth media (NGM) alone or NGM supplemented with conditioned media (CM; ratio 1/1), which were derived from cultured fibroblasts, VW-MSCs and Ctrl- and HOX-transduced fibroblasts, for 5 days. Whole-cell lysates were generated and analysed by Western blot for the indicated proteins.

### Migration assay

Migration of the cells was investigated via time-lapse microscopy for 8 h after IR. Therefore, human microvascular endothelial cells (HMEC) were grown to confluence, irradiated and a thin wound was introduced by scratching with a 10 µl pipette tip. Wound closure was determined for the different treatments by measuring the migration distance using ImageJ 1.47t (Wayne Rasband, National Institutes of Health, USA). Cell cycle phases were analyzed flow cytometry of detached cells 24 h after treatment using Nicoletti/propidium iodide staining (PI; 0.1% sodium citrate(w/v), 50 µg/mL PI (v/v) and 0.05% Triton X-100 (v/v)) as previously described [62]. Cells were stained for 30 min in the dark and subsequently measured with the flow cytometer FACS Calibur (BD, Heidelberg, Germany, FL2). Analysis was done using FlowJo software.

### Colony-forming unit (CFU) assay

Cells were plated at a density of 100, 250, 500 and 1000 cells per well (triplicates) in 6-well culture dishes. Medium was changed every 2 days. After 10 days of culture, cells were washed with PBS, fixed with 4% (w/v) paraformaldehyde, and subsequently stained with 0.05% Coomassie Brilliant Blue. Colonies (≥ 50 cells/colony) were counted. The survival curves were established by plotting the log of the surviving fraction [60].

### Statistical analysis

If not otherwise indicated, data were obtained from at least three independent experiments (*n* = 3). Mean values were calculated and used for the analysis of standard deviation (SD) or standard error (SEM). Statistical significance was evaluated by one- or two-way ANOVA followed by Tukey’s, Sidak’s or Bonferroni multiple comparisons post-test as indicated. Statistical significance was set at the level of *P* ≤ 0.05 (*P ≤ 0.05, **P ≤ 0.01, ***P ≤ 0.005, ****, ^#^*P* ≤ 0.001). Data analysis was performed with Prism 5.0 software (GraphPad, La Jolla, California). For the differential expression analyses, the post hoc Benjamini–Hochberg procedure to decrease the false discovery rate was used as described before [53]. Adjusted *P* values smaller than 0.05 were considered statistically significant.

## Results

### Generation of VW-typical MSCs by direct lineage conversion

**Fig. 1:**
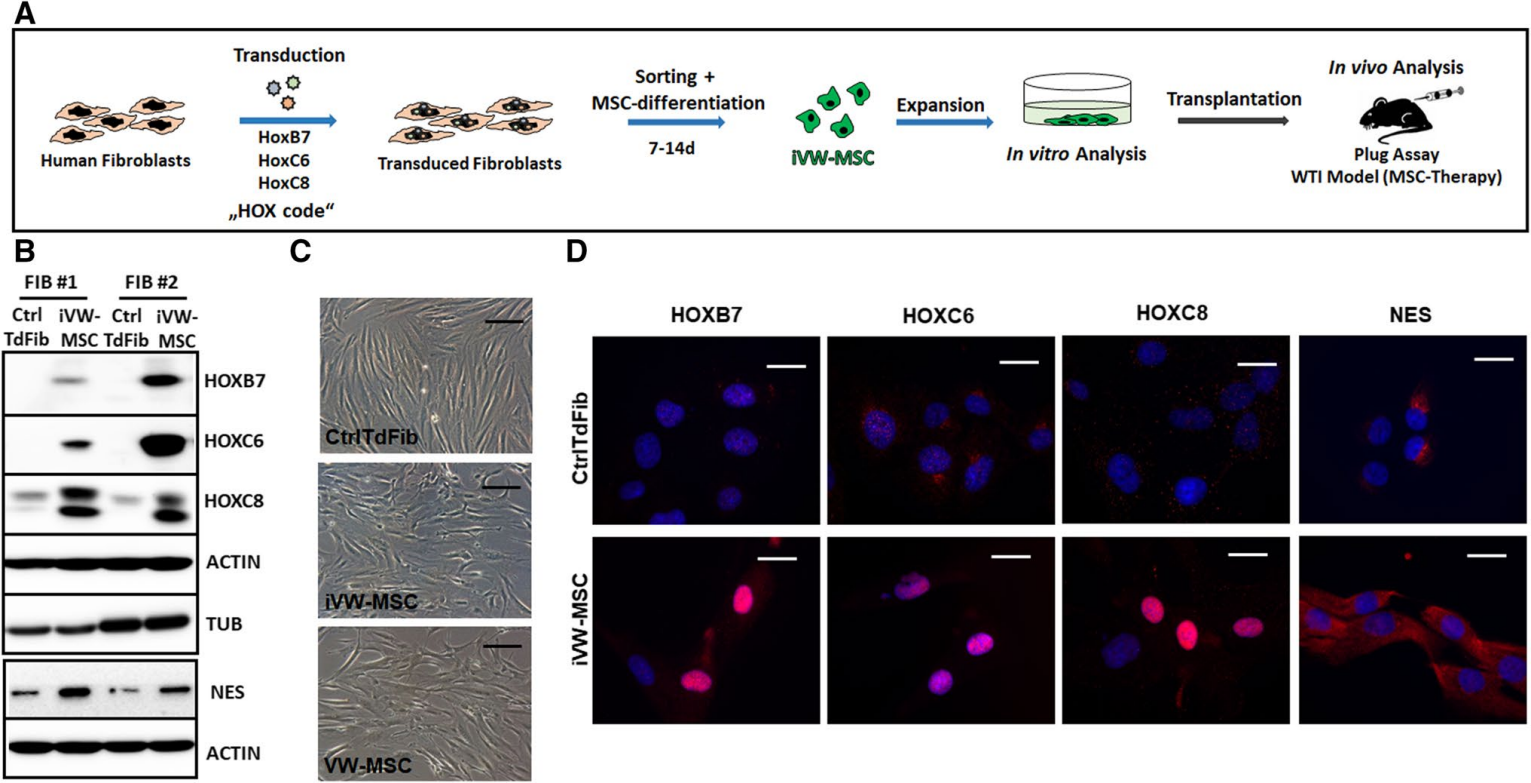
Induction of vascular wall-typical MSCs from adult human fibroblasts. **a** Experimental design for the induction of human MSCs. Primary human fibroblasts were transduced with a lentiviral SIN vector co-expressing the coding sequences of *HOXB7*, *HOXC6* and *HOXC8* and *Turquoise2 (Cyan)*, which are all co-translationally separated by 2A esterase moieties [48]. Two to four days after transduction the cells were sorted for cyan fluorescence and cultured in MSC medium. Generated MSCs (iVW-MSC) were characterized 14 days after induction when cells were sufficiently expanded. **b** Western blot analysis of total HOXB7, HOXC6 and HOXC8 protein expression levels as well as of Nestin (NES) were performed from whole cell lysates of HOX-transduced (iVW-MSC) and control fibroblasts (CtrlTdFib) 12–14 days after isolation of transduced cells. Representative blots from two transductions of fibroblasts from two independent donors are shown. Beta-actin (ACTIN) and alpha-tubulin (TUB) were included as loading controls. ^#^Fibroblasts derived from different healthy donors. **c** Representative phase contrast micrographs of cells 10–12 days after flow-cytometric sorting showed typical mesenchymal cell morphology. Ex vivo isolated hITA (human internal thoracic artery)-derived VW-MSCs were shown as control. **d** Sorted HOX-transduced and control fibroblasts were seeded on gelatine-coated cover-slips and HOXB7, HOXC6, HOXC8, and NES expression were detected by immunofluorescence using confocal microscopy. Representative photographs are shown

**Fig. 2:**
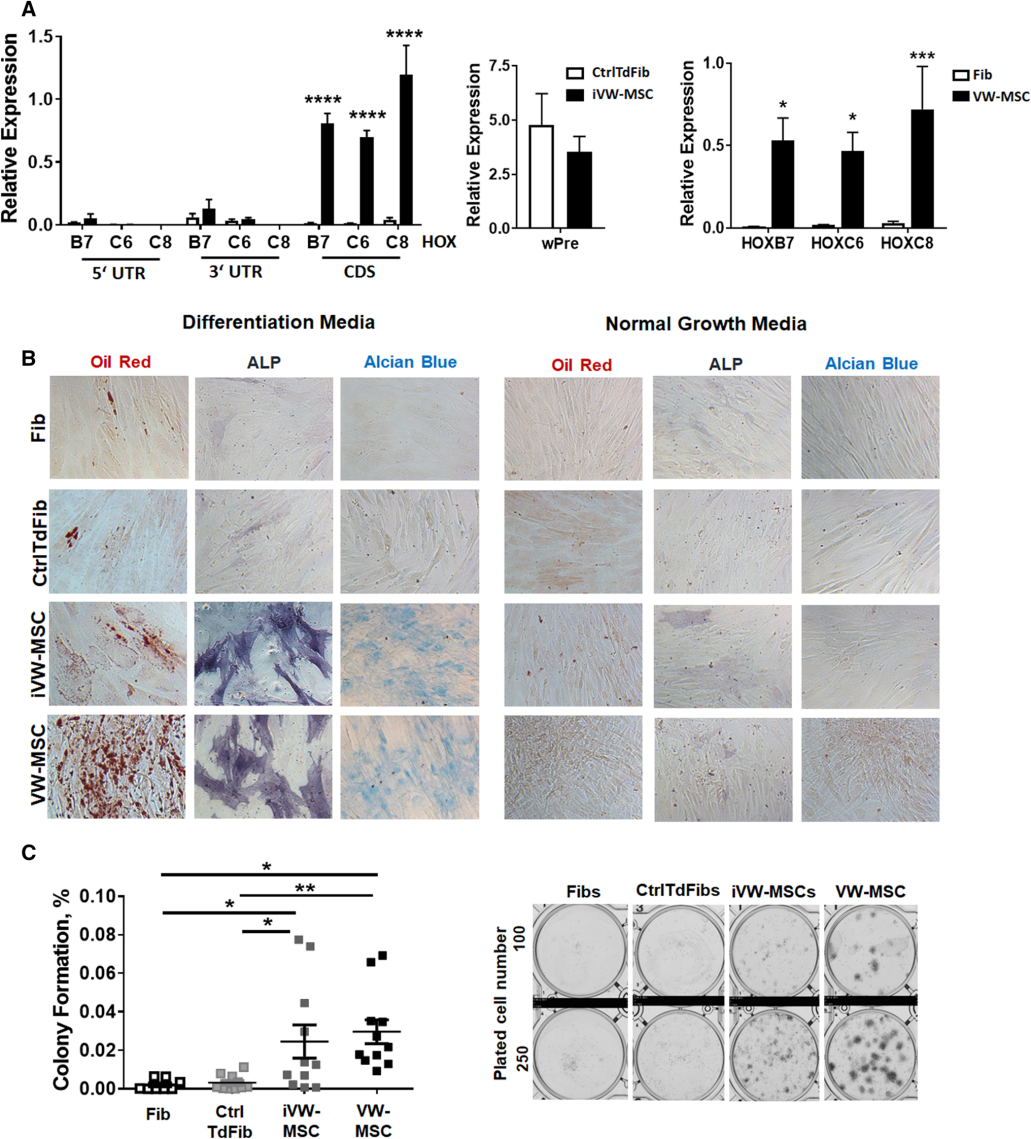
Characterization of control- and HOX-transduced fibroblasts (iVW-MSCs). **a** Relative amounts of transcripts of the indicated genes were determined by qRT-PCR in iVW-MSCs and control vector-transduced fibroblasts (CtrlTdFib) 12–14 days after transduction and flow-cytometry based cell isolation (biological replicates: *n* = 4–6 per group and gene; *P* by two-way ANOVA, followed by post hoc Tukey’s multiple comparisons test: ****P ≤ 0.001). For the detection of endogenous *HOX* gene expression, desoxy-oligonucleotide-primer pairs located in the 3′UTR as well as in the 5′UTR were used, which are not present in the retroviral expression vector containing only the coding sequence (CDS). For overall detection of retroviral vector expression, the woodchuck hepatitis virus posttranscriptional regulatory element (wPRE) was used. Differential HOX gene expression levels (CDS) in ex vivo isolated hITA (human internal thoracic artery)-derived VW-MSCs as compared to primary (non-transduced) fibroblasts (Fib) were included as positive and negative control. (biological replicates: *n* = 4–6 per group and gene; *P* by two-way ANOVA, followed by post hoc Tukey’s multiple comparisons test: *P ≤ 0.05; ***P ≤ 0.005). Relative transcript levels of analyzed genes were normalized to beta-actin mRNA (set as 1). **b** Verification of induced conversion into MSCs. FACS-purified iVW-MSCs and control fibroblasts were differentiated into adipocytes, osteocytes and chondrocytes, in vitro. Differentiation was observed within 14 days after induction of differentiation (DM) as shown by Oil red staining for detecting lipid droplets (red) in adipocytes, by histochemical NBT/BCIP staining for detecting alkaline phosphatase activity (ALP, black-purple) in osteocytes, or Alcian Blue staining (blue) for detecting acidic polysaccharides such as glycosaminoglycans in (e.g. the cartilage-specific proteoglycan aggrecan) in chondrocytes. Representative photographs are shown (biological replicates: *n* = 3–4). Magnification × 400. As control, respective cells were cultured in normal growth media (NGM). **c** CFU Assay. Control-transduced fibroblasts and iVW-MSCs were plated at low densities (100–1000 cells/well) in plastic culture dishes and subsequently cultured for 10 days. The lack of the colony-forming potential of primary fibroblasts as compared to hITA-derived VW-MSCs was confirmed by plating respective cells. Coomassie Brilliant Blue stained colonies were counted and the surviving fraction (colony formation) was calculated. *P* by two-way ANOVA, followed by post hoc Tukey’s multiple comparisons test: *P ≤ 0.05, **P ≤ 0.01 (biological replicates: *n* = 8–10 for each group)

To test whether vascular wall-specific MSCs can specifically be obtained by direct conversion of primary human fibroblasts in vitro, primary fibroblasts of different healthy donors were transduced with a self-inactivating (SIN) lentiviral vector co-expressing the coding sequences of *HOXB7*, *HOXC6* and *HOXC8* separated by 2A esterase elements, together with the gene encoding mTurquoise2 (mCyan-derived) fluorescent protein [48]. Transduced fibroblasts were sorted for mTurquoise2-fluorescence 2–4 days after transduction and further cultivated (Fig. 1 and Supplemental Figure S1). Expression of the endogenous and ectopic HOX proteins as well as of the MSC marker protein nestin was confirmed by Western blot (Fig. 1b). The process of transduction resulted in a more flattened MSC-typical phenotype as compared to the elongated cell morphology of control transduced fibroblasts (Fig. 1c). As visualized by immunofluorescence increased cytoplasmic as well as a prominent nuclear localization of the HOX proteins was observed in HOX-transduced fibroblasts (induced VW-MSCs, iVW-MSCs) (Fig. 1d). Steady-state endogenous and vector-mediated *HOX* transcription was further quantified by qRT-PCR (Fig. 2a). Significantly increased ectopic *HOXB7*, *HOXC6* and *HOXC8* mRNA expression levels were only detected in HOX-transduced fibroblasts. Of note, the classical MSC marker (CD90, CD73, CD105, CD146, STRO1) gene expressions as well as respective protein expressions were expressed in fibroblasts at similar levels as compared to ex vivo isolated human internal thoracic artery (hITA)-derived VW-MSCs, (Supplemental Figure S1), which resulted only in a further increase of the MSC marker expression levels by tendency in the generated VW-MSCs, derived from HOX-transduced fibroblasts. These induced VW-MSCs were termed now iVW-MSCs. Cell proliferation was not affected in iVW-MSCs as well as control-transduced fibroblasts that showed similar proliferative activities (Supplemental Figure S1).

**Fig. 3:**
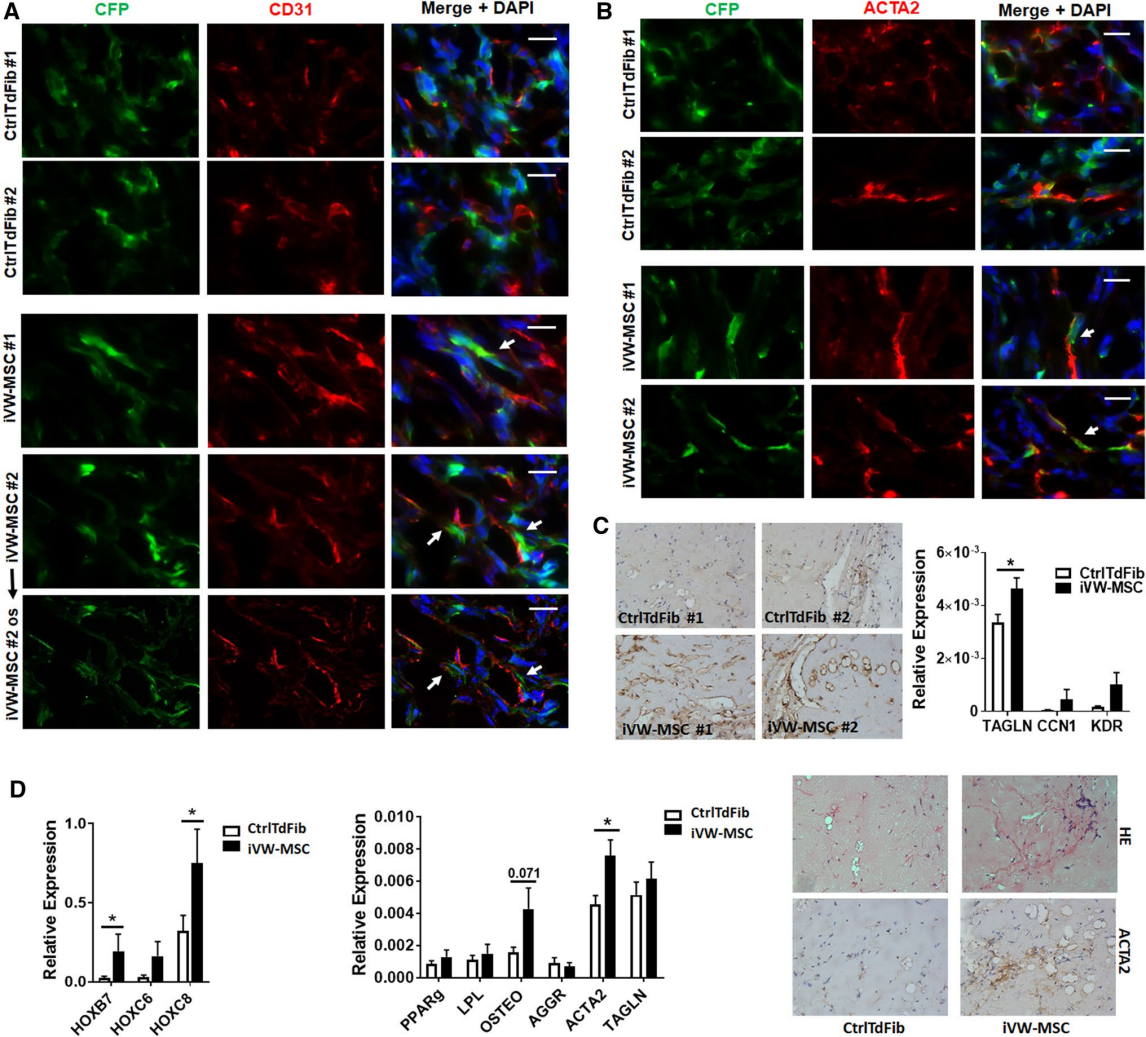
Contribution of iVW-MSCs to new vessel formation in vivo. **a** iVW-MSCs and control fibroblasts were grafted together with human endothelial cells (AS-M5) in Matrigel subcutaneously into NMRI nude mice for 14 days. Immunofluorescent analysis of re-isolated plug tissues was performed by confocal microscopy. Immunoreactivity to human CD31 is shown in red, expression of the reporter fluorescence protein Turquoise2 is depicted in green. Arrows point to the regular assembly of cyan-positive vascular wall MSCs that tightly surround the vessels formed by human endothelial cells. Optical sectioning (os) using structured illumination was used to further show the regular association mural cells derived from Turquoise2-positive iVW-MSCs. **b** Increased stabilization of newly formed blood vessels by Turquoise2(+) iVW-MSCs was further confirmed by double-immunofluorescence of the mural cell marker ACTA2 and CFP. Representative images of *n* = 4 independent experiments are shown. DAPI was used for nuclei staining. Scale bars: 50 µm. **c** Increased vessel formation and vascular stabilization were further confirmed by increased ACTA2 immunoreactivity of vascular structures in paraffin-sections of isolated plugs (left panel). qRT-PCR analysis of total RNA isolates (right diagram) generated from isolated plugs for the expression levels of the angiogenic endothelial marker gene vascular endothelial growth factor receptor 2 (KDR/VEGFR2) and the mural cell markers transgelin (TAGLN) and calponin (CNN). Biological replicates: *n* = 5–7 per group. *P* by two-way ANOVA, followed by post hoc Sidak’s multiple comparisons test: *P ≤ 0.05. **d** When control-transduced fibroblasts as well as iVW-MSCs were implanted alone (without human endothelial cells), and thus without an angiogenic stimulus, qRT-PCR analysis of indicated genes (*n* = 5–6), as well as histology and immunohistochemistry of ACTA2 confirmed the trend that iVW-MSCs stabilize vascular structures. qRT-PCR quantifications of indicated transcripts were shown as relative expression to beta-actin (set as 1). *P* by two-way ANOVA, followed by post hoc Sidak’s multiple comparisons test: *P ≤ 0.05

The propensity of iVW-MSCs to differentiate towards adipocytes, osteoblasts and chondrocytes was tested by plating and culturing the cells in appropriate differentiation media for additional 14 days (Fig. 2b and Supplemental Figure S2). Adipogenic, osteogenic as well as chondrogenic differentiation of iVW-MSCs was more efficient when the three *HOX* genes were ectopically expressed as confirmed by the quantification of the significantly increased expression levels of the differentiation marker genes peroxisome proliferator-activated receptor gamma (adipocytes), alkaline phosphatase and osteocalcin (osteocytes) and aggrecan (chondrocytes) upon differentiation (Supplemental Figure S2). In addition, the propensity for CFU (colony forming unit)-formation was significantly higher in iVW-MSCs as compared to the controls (Fig. 2c). To test whether the iVW-MSCs are able to contribute to the morphogenesis of functional blood vessels, in vivo, we subcutaneously transplanted the transduced cells either alone or together with endothelial cells in Matrigel into immune-deficient NMRI nude mice (Fig. 3). After 14 days Matrigel plugs were re-isolated and human CD31 and Turquoise 2 reporter-fluorescence was detected. Formation of new blood vessels within the plugs was demonstrated by the presence of vessels lined by CD31-positive endothelial cells. Turquoise2/HOX-positive cells were closely associated to these vessels displaying a flattened and elongated phenotype, thus indicating the potential differentiation of induced and co-implanted VW-MSCs towards vascular mural cells, e.g. pericytes and smooth muscle cells (Fig. 3a). Only single Turquoise2-positive control-transduced fibroblasts were detected close to the newly formed vascular structures, that might have resulted from the myogenic differentiation potential of the plastic donor cells (Fig. 3a). Compared to plugs containing control-transduced fibroblasts, plugs with iVW-MSCs showed a stronger vascularization and larger, more stabilized newly formed blood vessels (Fig. 3a–c). Fibroblast-derived MSC-differentiation into pericytes was further confirmed by co-immunostaining of the pericytes/smooth muscle cell marker smooth muscle actin (ACTA2) and the reporter protein Turquoise 2/CFP (Fig. 3b). The increased differentiation potential towards pericytes and smooth muscle cells of iVW-MSCs was further confirmed by increased ACTA2 immunoreactivity of vascular structures in paraffin-sections of isolated plugs, as well as by increased mRNA expression levels of the mural cell markers transgelin (TAGLN) and calponin (CNN) in respective plugs (Fig. 3c). An increased differentiation potential of iVW-MSCs towards stabilizing mural cells was even observed when iVW-MSC were implanted alone and thus without an angiogenic stimulus (Fig. 3d). To proof the non-tumorigenic potential, NMRI nude mice were subcutaneously injected with iVW-MSCs as well as control-transduced fibroblasts. No tumor growth was observed for all implanted cell charges following 60 days after tumor implantation (0/8 per HOX/Ctrl transduction each; transduced fibroblasts from two independent donors were investigated) (not shown).

Conclusively, the ectopic expression of three MSC-specific *HOX* genes promoted the in vitro differentiation of fibroblasts towards HOX-positive multipotent MSCs, and thus towards vascular wall-typical MSCs. Similar to ex vivo isolated VW-MSC, iVW-MSCs showed an increased colony-forming capacity and differentiation potential, which are specific important properties that discriminate MSCs from fibroblasts. In contrast, conventional stem cell properties such as plastic adherence and the expression of classical MSC markers (e.g. CD44, CD90, CD105) were unspecific for the MSCs as compared to the donor fibroblasts.

### Comparative global profiling of generated VW-MSCs

**Fig. 4:**
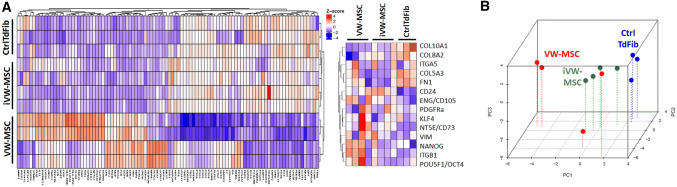
Global gene expression analysis. **a** Global transcriptome heatmaps (top 100 variant genes; left) of VW-MSCs, iVW-MSCs (HOX) and control-transduced fibroblasts as determined by microarray analysis. Classical MSC and fibroblast marker genes are additionally depicted in the small heatmap (right) (biological replicates as indicated: VW-MSC *n* = 4; HOX *n* = 4, Ctrl *n* = 3). **b** Principle component analysis of all differentially expressed genes (‘The Rohart MSC test’) further indicates that iVW-MSCs exhibit global gene expression profiles similar to those of hITA-derived VW-MSCs

Next, we investigated to what extent human iVW-MSCs resemble ex vivo isolated hITA-derived VW-MSCs. Therefore we compared the global gene expression and DNA methylation (DNAm) profiles of the iVW-MSCs to those of control-transduced fibroblasts and hITA-derived VW-MSCs (Fig. 4, Supplemental Figure S3). Global gene expression analysis showed that the gene expression profiles of ectopic *HOX*-expressing iVW-MSCs got a typical phenotype like the hITA-derived VW-MSCs, and thus were closely related to hITA-derived VW-MSC but distinct from control-transduced fibroblasts (Fig. 4a, b). The principle component analysis using the Rohart MSC signature genes [63] extracted from the global gene expression profiles confirmed that close relationship (Fig. 4b). Analysis of the DNAm profiles of the different cell preparations revealed a more diverse relationship (Supplemental Figure S3). Although the DNAm profiles of iVW-MSCs and hITA-derived VW-MSCs seem to come closer together, discrepancies were still prominent.

### The transcriptional signature of induced fibroblast-derived VW-MSCs ectopically expressing HOXB7, HOXC6 and HOXC8 confirms the acquisition of a MSC phenotype

**Fig. 5:**
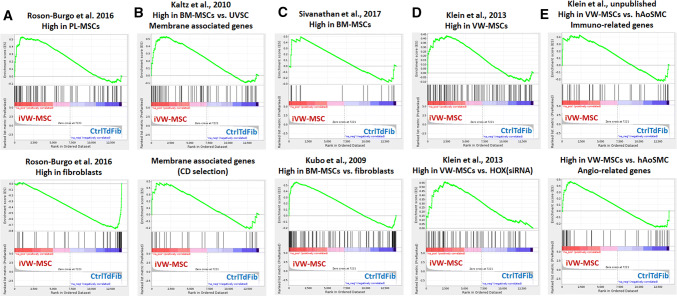
Gene Expression Profiling of iVW-MSCs. **a** GSEA was performed using gene sets specific for different MSCs. Genes highly expressed in human MSC derived from placental tissue (PL-MSC) as compared to human skin fibroblasts were used [64]. **b** Membrane-associated genes (top panel) and a CD selection (bottom panel) highly expressed in bone marrow-derived MSC (BM-MSC) as compared with stromal cells derived from umbilical veins (UVSC) were used [116]. **c** Genes highly expressed in untreated BM-MSC as compared to (pre-activated) IFN- treated BM-MSC (top panel) [117], as well as genes expressed selectively in BM-MSC compared with fibroblasts, osteoblasts, chondrocytes and adipocytes [118]. **d** Genes highly expressed in VW-MSC compared to VW-MSCs upon HOX silencing were used [49]. Top panel represents all significantly altered genes upon HOX gene silencing; bottom panel represents genes highly expressed in VW MSC as compared to those downregulated in VW-MSCs upon HOX silencing. **e** Genes highly expressed in VW-MSC as compared to human aortic smooth muscle cells (hAoSMC) were used (unpublished data set). cDNA microarray analysis using AffymetrixH DNA chips was performed to identify potential VW-MSC markers as compared to the mature vascular wall cells. Resulting significantly different expressed genes were grouped according to gene ontology and genes involved in the immune response (54 genes in total of which 34 genes were highly upregulated in VW-MSCs) as well as involved in angiogenesis (56 genes in total of which 22 genes were highly upregulated in VW-MSCs) were used as gene sets. Genes were drawn according to their rank from left (high expression in iVW-MSCs) to right (high expression in Ctrl) and gene sets plotted on top with each black bar representing a gene. The enrichment score is plotted in on the vertical axis. Specific gene sets were listened in the Supplementary Table S4

To further confirm if iVW-MSCs displayed a gene expression signature corresponding to classical and well-known MSCs we performed Gene Set Enrichment Analysis (GSEA) (Fig. 5 and Supplementary Table S4). We first used the gene sets specific for MSCs derived from human placental tissue (PL-MSCs) as compared to primary skin fibroblasts as previously described [64]. This GSEA uncovered that the iVW-MSCs with ectopic expression of the HOX-code most closely resembled a transcriptional signature of PL-MSCs while the control transduced fibroblasts exhibited the typical signature of (skin) fibroblasts (Fig. 5a and Supplementary Table S4). To further refine the analysis, we asked how closely these iVW-MSCs resembled classical MSCs derived from the bone marrow (BM-MSCs). Again, GSEA indicated a closer relationship of iVW-MSCs to BM-MSCs than control transduced fibroblasts (Fig. 5b, c, and Supplementary Table S5). We finally asked whether the transcriptome of iVW-MSCs rather displayed the already identified VW-MSC HOX signature using gene sets from VW-MSCs either control or transfected with HOX siRNA [49]. Again, here GSEA clearly indicated the similarity between iVW-MSCs and ex vivo isolated human VW-MSCs (Fig. 5d and Supplementary Table S4). With respect to the suggested therapeutic potential of iVW-MSCs, we compared the transcriptional signature of generated cells to gene sets which were derived from the comparison of ex vivo isolated VW-MSCs to mature human aortic smooth muscle cells (hAoSMC; unpublished gene set). Herein, genes sets were used which comprise genes related to an immunomodulatory function as well as genes involved in angiogenesis (Fig. 5e and Supplementary Table S4). Of note, the iVW-MSCs were highly similar with the ex vivo isolated human VW-MSCs, further supporting our notion that these cells were (i) characterized by a MSC transcriptional phenotype similar to that of VW-MSCs and suggesting that (ii) these cells could be used for therapeutic approach.

### Regulated expression of HOXB7, HOXC6 and HOXC8

**Fig. 6:**
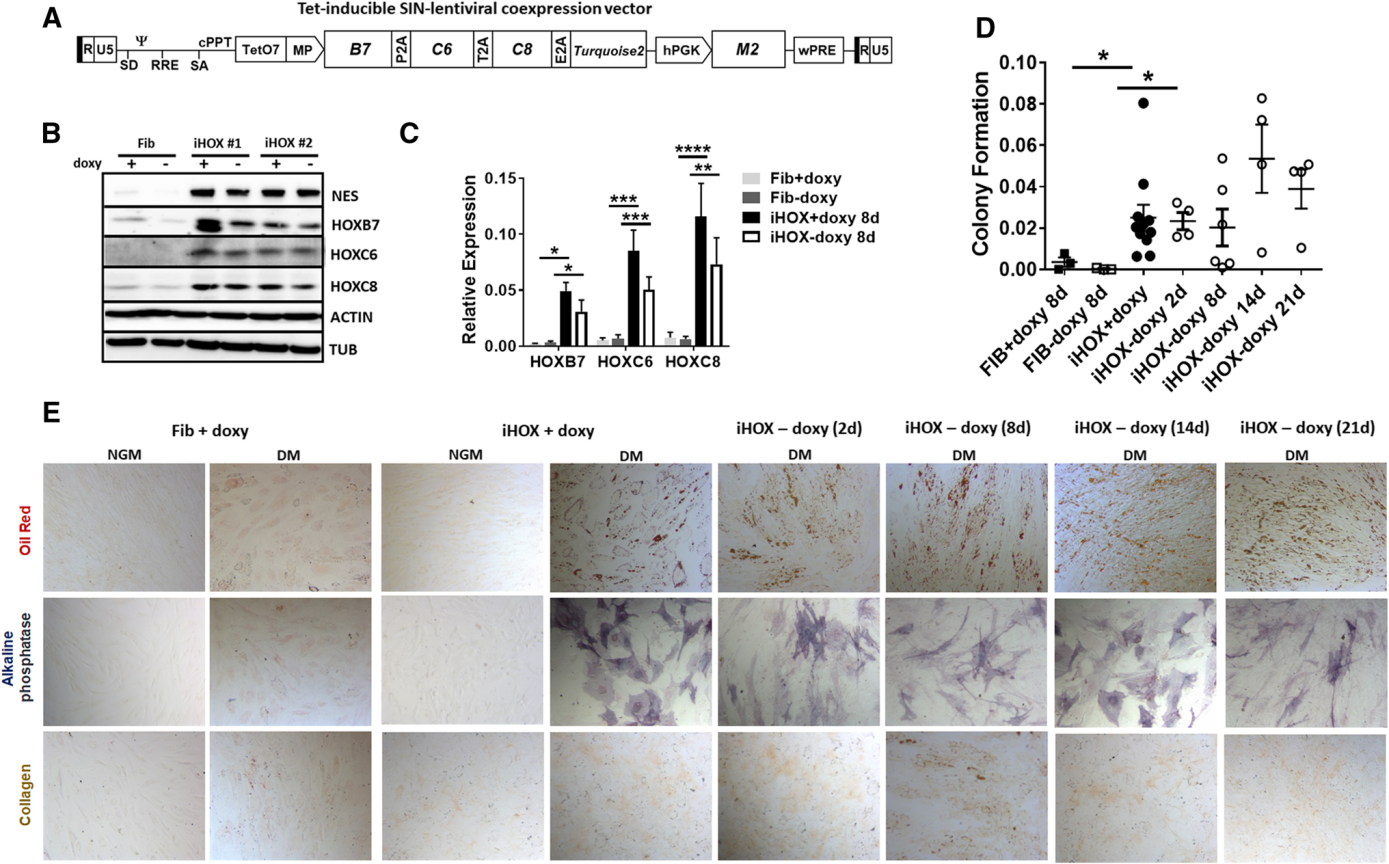
Regulated expression of HOXB7, HOXC6 and HOXC8. To address the question whether the reprogrammed state of the generated VW-MSCs is stable or not, a doxycycline-inducible retroviral expression vector (“all-in-one tet-on” system) for controllable expression of the HOX co-expression cassette was generated. **a** Scheme of the self-inactivating lentiviral vector for doxycycline-inducible expression of the coding sequences of *HOXB7*, *HOXC6* and *HOXC8* separated by *2A esterase* elements together with the gene encoding the reporter TURQUOISE2 fluorescent protein. **b** Primary human fibroblasts were transduced with the doxycycline inducible SIN vector encoding for the HOX code. Transduced cells (iHOX) were treated with doxycycline (0.2–0.5 µg/ml) 48 h after transduction, sorted for cyan fluorescence after expansion (additional 4–6 days) and then cultured in MSC medium. Mock-transduced fibroblasts either doxycycline-treated or not were used as control. Western blot analysis of total HOXB7, HOXC6 and HOXC8 protein expression as well as of Nestin (NES) marker protein expression was performed from whole cell lysates of HOX-transduced and control fibroblasts with or without doxycycline withdrawal for 4 days. Beta-actin (ACTIN) and alpha-tubulin (TUB) were included as loading controls. ^#^Fibroblasts derived from (2) different healthy donors. No changes of the doxycycline-induced HOXB7, HOXC6 and HOXC8 protein expression levels were detectable 4 day after doxycycline withdrawal. **c** Relative amounts of transcripts of the introduced HOX genes were further determined by qRT-PCR 8 days after doxycycline withdrawal (biological replicates: *n* = 4 per group and gene; *P* by two-way ANOVA, followed by post hoc Tukey’s multiple comparisons test: *P ≤ 0.05; **P ≤ 0.01; ***P ≤ 0.005; ****P ≤ 0.001). Relative transcript levels of analyzed genes were normalized to beta-actin mRNA (set as 1). No significant down-regulation of the doxycycline-induced HOXB7, HOXC6 and HOXC8 mRNA expression levels were detectable 8 day after doxycycline withdrawal, although the respective HOX expression levels trend to decline. **d** CFU Assay. The colony-forming potential of doxycycline-HOX-induced VW-MSCs as compared to primary fibroblasts was investigated by plating respective cells at low densities and subsequently culturing for 10 days in the presence or absence of doxycycline for the indicated days. Quantification of the surviving colonies revealed that 2–21 days after doxycycline-withdrawal generated VW-MSCs (iHOX) still possess colony-formation potential. **e** Verification of doxycycline-induced conversion into MSCs. FACS-purified, iHOX-transduced and control doxycycline-treated fibroblasts were differentiated into adipocytes, osteocytes and chondrocytes at the indicated time points after doxycycline removal. Differentiation was observed within 14 days after induction of differentiation (DM) as shown by Oil red staining (adipocytes), by histochemical staining for alkaline phosphatase (ALP) visualizing osteocytes, or by immunocytochemistry for cartilage-specific collagen type II expressions (chondrocytes). Representative photographs are shown. Magnification × 400. As control, respective cells were cultured in normal growth media (NGM). Of note, 14–21 days after doxycycline-withdrawal generated VW-MSCs (iHOX) still possessed trilineage differentiation potential

Prior to testing the therapeutic potential, we wanted to address the question whether the reprogrammed state of the iVW-MSCs is stable or not (Fig. 6). Therefore, we constructed a doxycycline-inducible SIN-lentiviral expression system for a controlled expression of *HOXB7*, *HOXC6* and *HOXC8* (Fig. 6a). Primary human fibroblasts were transduced with this vector, treated with doxycycline for 48 h after transduction, sorted for cyan fluorescence and expanded as iHOX cells. Western blot analysis of total HOXB7, HOXC6 and HOXC8 as well as NES marker protein expression was performed from whole-cell lysates of HOX-transduced and control fibroblasts after the withdrawal of doxycyclin for four days and revealed a continued HOX protein expression after that time (Fig. 6b). qRT-PCR analysis further revealed no significant down-regulation of the doxycycline-induced HOXB7, HOXC6 and HOXC8 mRNA expression levels 8 days after doxycycline withdrawal, although the respective HOX expression levels trend to decline (Fig. 6c). In line, MSC-specific differentiation capabilities and the colony-forming potential of generated iHOX-VW-MSCs remain unchanged and significantly increased as compared to control-treated primary fibroblasts (Fig. 6d). Up to 21 days after doxycycline removal we were not able to detect a significant downregulation of HOX protein levels and a reduction in clonogenecity and differentiation capabilities, which was due to the induction of endogenous HOX gene expressions (Fig. 6d, e and Supplementary Figure S4). As compared to donor fibroblasts, adipogenic, osteogenic as well as chondrogenic differentiation of iHOX-VW-MSCs was more efficient when the three *HOX* genes were ectopically expressed (Fig. 6e).

Conclusively, these results suggest that after a long induction, the induced expression of the HOX code resulted in a stable VW-MSC phenotype that, in line with the results presented above, resulted in elevated colony-forming capacities and differentiation potentials as important properties to discriminate MSCs from fibroblasts.

### Therapeutic potential of generated fibroblast-derived VW-MSCs

**Fig. 7:**
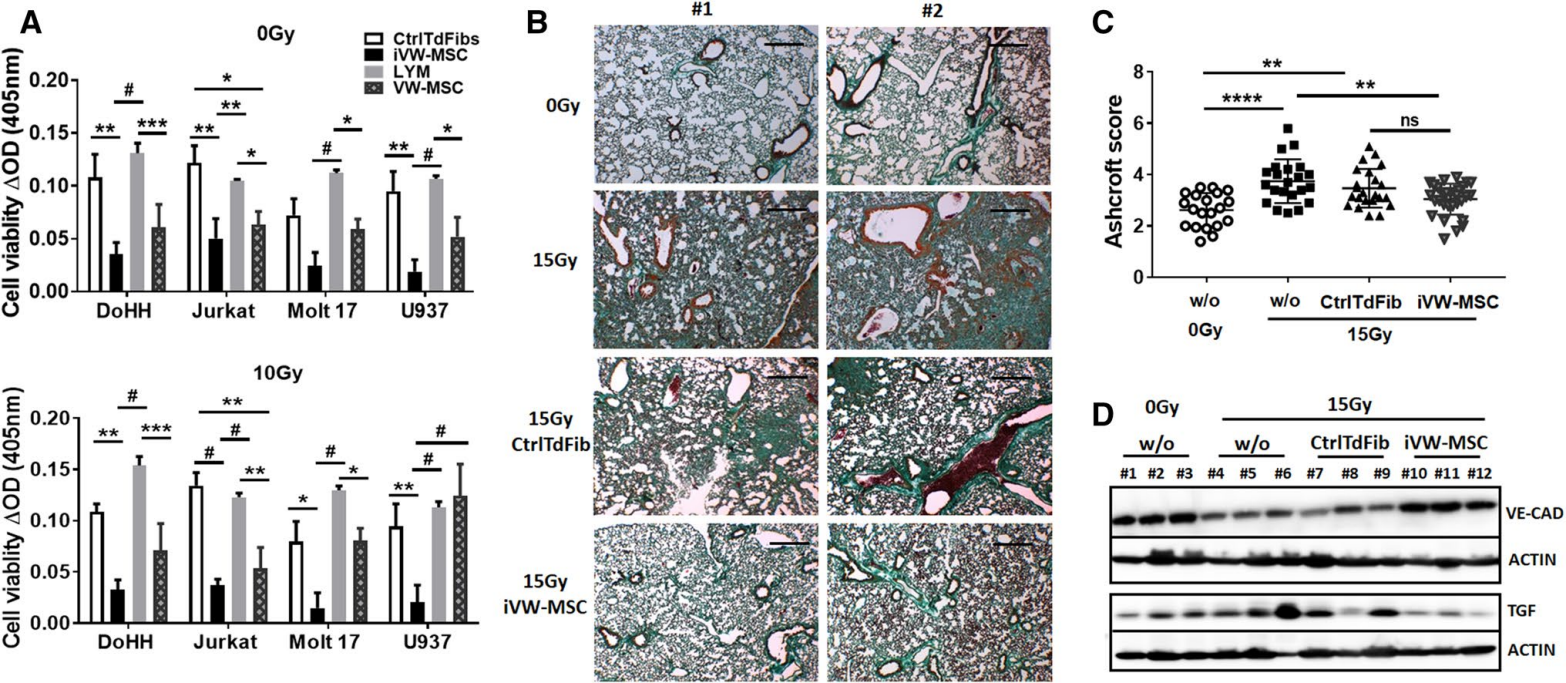
Therapeutic potential of iVW-MSCs. **a** Verification of lymphocyte proliferation inhibition: HOX-transduced and control fibroblasts were co-cultured with different lymphoma cells (DoHH2, Jurkat, MOLT17, and U937) for 24 h. VW-MSCs were used as positive control. Cell-cycle arrested, irradiated (10 Gy, 24 h prior to co-culture) fibroblasts/VW-MSCs were used to exclude possible effects mediated by their proliferation. (bottom diagram). Cell proliferation was determined using a WST-1 reagent-based tetrazolium reduction assay and related to proliferation of lymphoma (LYM) cells alone (biological replicates: *n* = 5–6 per group and lymphoma cell line; *P* by two-way ANOVA followed by post hoc Tukey’s multiple comparisons test: *P ≤ 0.05; **P ≤ 0.01; ***P ≤ 0.005; ^#^*P *≤ 0.001). **b** Therapeutic applied iVW-MSCs limit radiation-induced lung fibrosis and endothelial cell loss: C57BL/6 mice were left untreated or received a 15 Gy whole thorax irradiation (WTI). Single cell suspensions of cultured iVW-MSCs or control-transduced fibroblasts (CtrlTdFib) (0.2 × 10^6^ cells) were intravenously transplanted via tail vein injection into WTI mice 24 h after irradiation. 25 weeks later, sections of paraffin-embedded lung tissue were generated Masson’s Goldner Trichrome staining was performed to visualize the connective tissue. Sham irradiated (0 Gy) animals served as control. Representative light microscopy images from two different experiments (#1, #2) are shown (scale bar = 100 µm). **c** Quantification of lung fibrosis was done by determining the Ashcroft scores blinded to the genotype and treatment conditions. Data are presented as mean ± SEM. **P ≤ 0.01; ****P ≤ 0.001 by one-way ANOVA followed by post hoc Tukey’s test (*n* = 15–25 mice per group). **d** Endothelial VE-Cadherin (VE-CAD) and TGFβ expressions were analyzed in whole protein lysates from mice lungs using Western blot analysis 25 weeks post irradiation (*n* = 5–6 per group). Beta-actin was included as loading control. Representative blots from the lungs of three different mice per group were shown

The potential of iVW-MSCs to mediate immunomodulation was investigated by testing their ability to inhibit lymphocyte proliferation using an allogeneic mixed lymphocyte reaction with different human non-adherent lymphoma cells as mitogens (MOLT17, DoHH2, Jurkat, and U937). Cell-cycle-arrested, irradiated (10 Gy) iVW-MSCs and control-transduced cells, as well as VW-MSCs as a positive control, were also used to determine background proliferation during the measurements. Lymphocyte proliferation was determined after 24 h of co-culture. VW-MSCs, as well as iVW-MSCs significantly suppressed the proliferation of the different lymphoma cells (Fig. 7a). Next, we investigated whether therapeutic applied iVW-MSCs were able to reduce radiation-induced lung fibrosis and endothelial cell loss in a whole thorax irradiation (WTI) model of radiation-induced lung disease, established by our group [51, 60] (Fig. 7b–d and Supplemental Figure S5). For this, single cell suspensions of cultured iVW-MSCs as well as of control-transduced fibroblasts were intravenously transplanted into the tail vein of irradiated (15 Gy WTI) or untreated C57BL/6 mice, 24 h after irradiation. Peripheral blood cell analysis within the inflammatory phase at 3 weeks after irradiation revealed decreased total leukocyte numbers upon radiation, whereas treatment with iVW-MSCs normalized these levels by tendency. Of note, a radiation-induced increase of monocyte and granulocyte numbers was normalized in treated animals (Supplemental Figure S5). Analysis of Turquoise2/HOX expression in isolated lung sections revealed that nearly none of the therapeutic applied cyan-positive cell homed to the injured lung tissue (not shown). However, circulating iVW-MSCs could still be detected in peripheral blood several weeks after transplantation (Supplemental Figure S5). We then analyzed the impact of implanted cells on fibrosis and endothelial cell loss (Fig. 7c, d). Development of radiation-induced fibrosis was investigated 25 weeks after WTI on sections of paraffin-embedded lung tissue and staining by Masson´s Goldner Trichrome (connective tissue), and revealed that the therapeutic applied iVW-MSCs significantly limited fibrosis development (Fig. 7b, c). Treatment with iVW-MSCs significantly also attenuated the synthesis of the pro-fibrotic cytokine TGF-β1, as detected in homogenized whole lung tissue by Western blot analysis (Fig. 7d). Interestingly, radiation-induced endothelial cell loss was significantly limited in animals which were treated with iVW-MSCs as revealed by restored endothelial VE-Cadherin expressions in WTI-lungs 25 weeks post irradiation (Fig. 7d). We have to emphasize that the implanted human MSCs or factors derived thereof may not have been as effective as the endogenous mouse-specific factors in terms of tissue-regeneration and/or -protection, most likely due to species reasons within this in vivo xenotransplantation model.

**Fig. 8:**
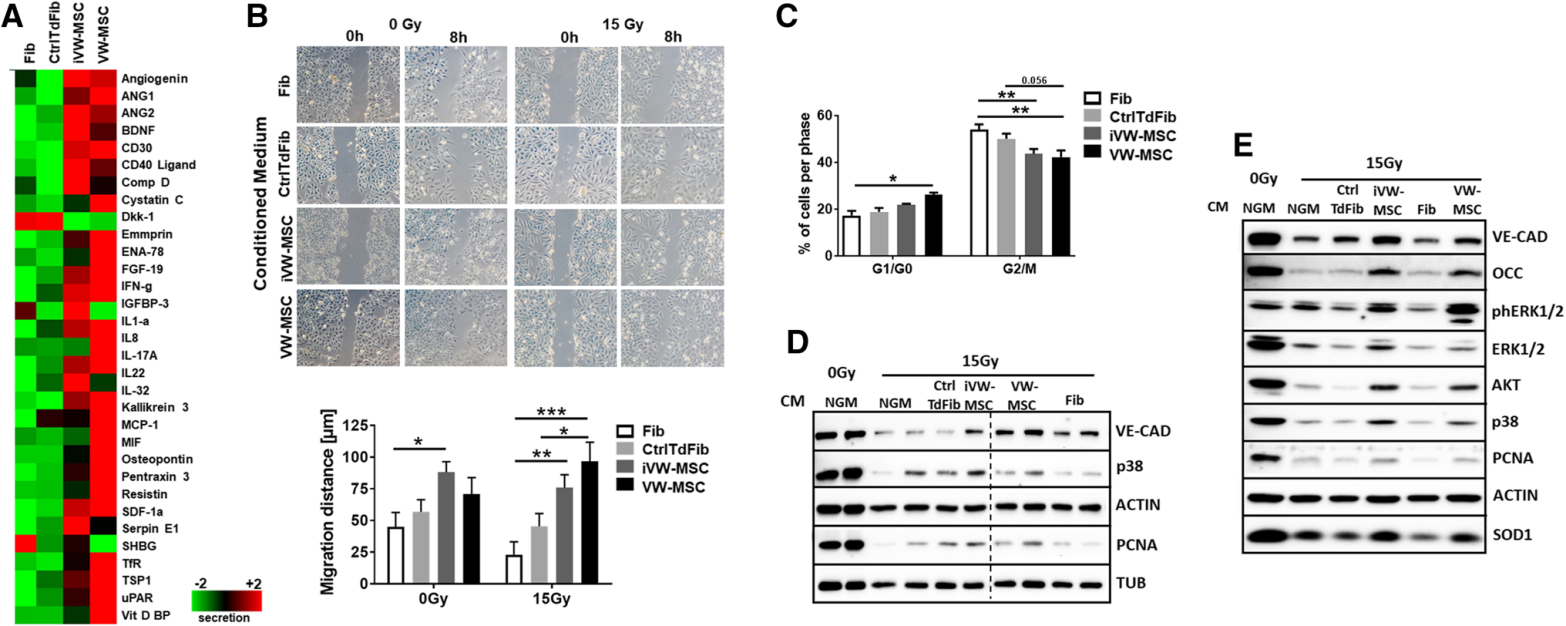
VW-MSC-specific paracrine action for mediating radio-protection especially of endothelial cells. **a** A membrane-based sandwich immunoassay (Proteome Profiler Human XL Cytokine Array Kit, RnD Systems) was used for the parallel determination of the relative levels of selected human cytokines and chemokines in cell culture supernatants (conditioned medium, CM) of fibroblasts, VW-MSCs, Ctrl-transduced fibroblasts (CtrlTdFib) and iVW-MSCs. Equal protein amounts were run on each array. Profiles of detected signals were quantified by densitometry and related to the reference signal (set as 1). Results from one representative experiment out of two independent biological replicates (supernatants) measured in duplicates each were presented as heatmap. **b** Endothelial cell migration was investigated after irradiation and subsequent introduction of a thin wound in confluent monolayers by scratching with a pipette tip. Wound closure was determined for the different supernatant treatments by measuring the migration distance after 8 h. Data are shown as mean ± SEM of three independent experiments measured in duplicates each. ***P ≤ 0.001, **P ≤ 0.01 *P ≤ 0.05 by two-way ANOVA followed by post hoc Sidak’s test. **c** Cell cycle phases (G0/G1 and G2/M) of irradiated endothelial cells and subsequent conditioned medium (CM) treatment was further analyzed after 24 h. Data are shown as mean ± SEM of three independent experiments measured in duplicates each. **P ≤ 0.01 *P ≤ 0.05, by two-way ANOVA followed by post hoc Tukey’s test. **(D)** Cultured endothelial cells were exposed to irradiation with 15 Gy, subsequently cultured in normal growth media (NGM) or NGM supplemented with CM of fibroblasts, VW-MSCs, Ctrl-transduced fibroblasts (CtrlTdFib) and iVW-MSCs. Expression levels of the indicated proteins were analyzed in whole protein lysates using Western blot analysis at 96 h after radiation and subsequent growth factor treatment. Representative blots from four different experiments are shown (*n* = 3). **e** Cultured normal lung tissue fragments embedded in growth factor reduced matrigel were exposed to irradiation with 15 Gy, subsequently cultured in normal growth media (NGM) alone or NGM supplemented with conditioned media (CM; ratio 1/1), which were derived from cultured fibroblasts, VW-MSCs, Ctrl-transduced fibroblasts and iVW-MSCs. Expression levels of the indicated proteins were analyzed in whole protein lysates using Western blot analysis at 5 days after radiation and subsequent CM treatment. Representative blots from four different experiments are shown (*n* = 4)

These results indicated that soluble factors, or products derived from VW-MSCs, modulate the immune response and suggest that converted/induced MSCs are able mediate immunosuppression in a manner similar to primary MSCs, at least in vitro. Therefore, we investigated growth factors and cytokines, which were extracted from the global gene expression profiles (Supplemental Figure S6). Ex vivo isolated VW-MSCs as well as iVW-MSCs expressed immunosuppressive cytokines, e.g. indoleamine-2,3-dioxygenase (IDO1) and histocompatibility antigen class I–G/, also known as human leukocyte antigen G (HLA-G), as well as the established exosome markers CD9, CD63, and ADAM10. Each of these markers were expressed higher than in control-transduced cells suggesting that MSC-secreted exosomes may be involved in immunosuppression (Supplemental Figure S6). To gain more insight how iVW-MSCs are involved in tissue integrity and/or foster tissue regeneration as compared to fibroblasts, we further performed multiplex antibody arrays to detect cytokines, chemokines, and acute-phase proteins in conditioned medium generated from cultured fibroblasts, VW-MSCs and Ctrl-transduced fibroblasts and iVW-MSCs (Fig. 8). The array membranes incubated with the conditioned media of the cells featured a predominant hybridization signal for prominent angiogenic factors (e.g. angiogenein, ANG1/2) and immunomodulating cytokines (e.g. interleukins, MCP1, INF-g and SDF) in control VW-MSCs and iVW-MSCs (Fig. 8a). Concurrently, the cytokine secretion profile confirmed that iVW-MSCs were very similar to hITA-derived VW-MSCs.

To test our findings about the radioprotective and/or tissue-regeneration fostering and, also paracrine effects of iVW-MSCs, we used endothelial cell cultures and ex vivo tissue cultures of human normal lung tissue (Fig. 8b–e). The assumed protective action of factors secreted from VW-MSCs was first corroborated on cultured endothelial cells by analyzing their migration and proliferation potential upon radiation and subsequent conditioned media treatment that were derived from cultured fibroblasts, VW-MSCs, Ctrl-transduced fibroblasts and iVW-MSCs (Fig. 8b, c). Interestingly, treatment with iVW-MSC supernatants rescued the radiation-induced reduction in migration (Fig. 8b) and limited the radiation induced M/G2-arrest of cultured endothelial cells (Fig. 8c and Supplemental Figure S7). Radiation led further to a downregulation of VE-CAD expression in cultured endothelial cells, whilst MSC-derived CM limited the reduction (Fig. 8d). Expression levels of the proliferation marker Proliferating-Cell-Nuclear-Antigen (PCNA) were also restored in VW-MSC CM-treated endothelial cells [60, 62].

The paracrine-based radioprotective action of iVW-MSCs on the vascular compartment was further confirmed in human lung tissue cultures upon radiation and subsequent VW-MSC factor treatment (Fig. 8e). Fresh lung specimens were dissected and embedded in matrigel prior to irradiation with 15 Gy, with or without subsequent culture in normal growth media (NGM) alone or NGM supplemented with the different conditioned media (Fig. 8e). Western blot analyses of total protein extracts confirmed that radiation fostered a (well-known) downregulation of VE-CAD expressions and of the associated junctional protein occludin in cultured lung tissues [62], whilst treatment with conditioned media derived from iVW-MSCs and hITA-derived VW-MSCs clearly limited endothelial cell loss. We then confirmed the restoration of the radiation-induced impairment of endothelial cell signaling pathways activated upon CM treatment. Mitogen-activated protein kinase (MAPK) pathways including extracellular signal-regulated kinases 1 and 2 (ERK1/2) and p38 MAPK were restored upon treatment with VW-MSC derived paracrine signals. In addition, the radiation-induced reduction of the antioxidant enzyme superoxide dismutase 1 (SOD1), a MSC-secreted factor involved in radioprotection, adoptively transferred from MSCs [60], was restored in the tissue cultured, which were treated with VW-MSC CM (Fig. 8e). Thus, iVW-MSCs and MSC-derived factors were able to counteract radiation-induced late adverse effects of vascular damage and endothelial cell loss. The high activity of vascular wall-derived MSCs for radioprotection may be due to their tissue-specific action.

Taken together, our results strongly suggest that ectopic expression of VW-MSC-specific HOX genes promotes the conversion of fibroblasts to therapeutic active, vascular wall-typical, multipotent MSCs.

## Discussion

### iVW-MSCs-specific features of lineage converted fibroblasts

The direct conversion of cells by the introduction of target cell-type specific transgenes is a promising and widely used strategy to switch cellular identities [65, 66]. Direct cell fate conversion bypassing pluripotency could be achieved by transient, ectopic expression of cell-type specific transcription factors, miRNAs or using of epigenetic modifiers, [67–70]. Ectopically expressed lineage determining transcription factors were already shown to foster direct lineage conversions [71–73]. Herein, fibroblasts have been successfully converted into cells of other germ layers, such as neurons, hepatocytes, osteoblasts, and cardiac-like myocytes [47, 74–78]. Due to the lack of identified tissue-specific transcription factors in MSCs in terms of being master regulators, it was difficult to produce induced MSC up to now. Here, we directly converted fibroblasts towards vascular wall-typical MSCs (iVW-MSCs) by ectopic expression of a *HOX*-code, namely *HOXB7*, *HOXC6* and *HOXC8.* These highly VW-MSC specific genes were previously defined by us [48, 49]. Generated iVW-MSCs displayed classical multipotent characteristics as defined by ISCT [79] in vitro, and selectively associated with vascular structures in matrigel plug assays in vivo, thus likely representing true VW-MSCs. However, the conventional MSC properties such as plastic adherence and the expression of CD44, CD90, CD73 and CD105 turned out to be unspecific for phenotypic characterization of MSC as compared to fibroblasts, indicating that there is an urgent need in identifying additional cell type-specific markers. In general, MSCs show a fibroblast-like morphology, whereas fibroblasts are defined as non-endothelial, non-epithelial, and non-hematopoietic adherent cells of mesenchymal origin. These striking similarities were further underlined by the concept of cell plasticity that is also reported to pertain to fibroblasts, as mouse embryonic fibroblasts exhibited a greater plasticity than MSCs and, therefore, were suggested to be closer to embryonic stem cells [80]. As fibroblasts and MSCs share the mesenchymal phenotypes and even to a certain extent their gene expression profiles, the colony-forming capacity and differentiation potential turned out to be the specific important properties that discriminate MSCs from fibroblasts [81–84]. In line with these findings we demonstrated here that generated iVW-MSCs displayed significantly increased clonogenicity, and had increased capacity to differentiate into chondrocytes, osteocytes and adipocytes. In addition, iVW-MSCs showed the VW-MSC-typical potential to differentiate into vascular mural cells, namely pericytes and smooth muscle cells, finally resulting in vascular stabilization. Thus, based on the expression pattern of *HOX* genes *HOXB7*, *HOXC6* and *HOXC8* as well as the cell-type-specific features of VW-MSCs, the identified *HOX*-code could be one additional characteristic to discriminate VW-MSCs from phenotypical similar cells like fibroblasts as reported here and other vascular cells as previously reported [32, 49].

### The transcriptional profile of iVW-MSCs

The combination of the *HOX* genes *HOXB7*, *HOXC6* and *HOXC8* can further be considered as master regulator to directly convert fibroblasts into iVW-MSCs. The general MSC phenotype of iVW-MSCs was confirmed here by MSC-typical transcriptional signatures using GSEA as well as the Rohart MSC test that accurately distinguished MSC from non-MSC samples by a specific gene signature that is shared by a wide-variety of MSC [63]. However, stromal cell populations derived from various tissues are different, which may result in heterogeneity within the mesenchymal phenotype. We show here by comparative global gene expression profiling that iVW-MSCs were closely related to hITA-derived VW-MSC, although the proximity to the dermal fibroblasts as donor material was still given. Isolated cells from different origins seem to keep expression ‘memory’ of source-specific genes that may travel along during the differentiation process [85]. The cell’s signature itself further dependents on the quality of the MSC used, which is critically influenced by the donor age, sex and life style, as well as isolation and cultivation methods [63]. In particular, it has to be considered that cells derived from individuals resemble their age. As compared to the young counterparts, fibroblasts from (very) old individuals display a typical transcriptional signature, shortened telomeres and dysfunctional mitochondria as well as higher levels of oxidative stress [86]. For example, neurons generated by direct conversion of fibroblasts from human donors across a broad range of ages revealed that important aging-related signatures were maintained in the desired target cells [87]. Reprogramming of fibroblasts of very old donors to the pluripotent state via the addition of the pluripotency genes NANOG and LIN28 to the Yamanaka reprogramming cocktail resulted in a widespread rejuvenation of generated iPSCs including epigenetic remodeling and expansion of telomeres [88]. This rejuvenation was preserved when iPSCs were differentiated back to fibroblasts. Thus, age-associated transcriptional signatures of the donor fibroblast will be maintained after the direct conversion to our iVW-MSCs. In addition, the difference in the age of fibroblast donors as compared to the age of the patients donating hITA and respective VW-MSCs might adversely influence the clustering of fibroblast-derived iVW-MSCs and hITA-derived VW-MSCs, as patient undergoing bypass surgery were usually of advanced age.

### The therapeutic potential of iVW-MSCs

With respect to their therapeutic potential, the generated iVW-MSCs suppressed lymphocyte proliferation, in vitro, and protected mice against radiation-induced pneumopathy. In previous studies, we already demonstrated that therapeutic applied VW-MSCs mediated radioprotection, in particular of the vascular compartment, predominately by a paracrine mechanism of action [51, 60, 62]. Herein, applied VW-MSC limited the radiation-induced activation of the usually quiescent lung endothelial cells, which then reduced radiation-induced immune cell infiltration (inflammation) and endothelial cell apoptosis [51, 60, 62]. Here we report that iVW-MSCs were particularly well suited to address and mitigate vascular impairments in a similar manner. iVW-MSC-derived factors were able to limit the radiation-induced vascular damage as revealed by (i) reduced inflammation and reduced endothelial cell loss that was accompanied by limited fibrosis progression in a mouse model of radiation-induced pneumopathy, (ii) limited endothelial impairments in ex vivo cultured human lung tissues, and (iii) reduced G2/M arrest as well as reduced cell migration of cultured endothelial cells. Therefore, the direct in vitro conversion of fibroblasts towards iVW-MSCs may be an efficient strategy for the treatment of diseases associated with vascular damage and remodeling, e.g. hypertension, ischemic diseases, vascular lesions, or irradiation [89–92]. For therapies based on MSCs, safety and efficacy have been proven in the last years. The formation of tumors was almost completely ruled out due to the generally limited differentiation potential of adult stem cells. The directed generation of multipotent stem cells of mesenchymal nature in vitro is an extremely promising approach for a number of therapeutic applications because single clones harboring vector integrations at safe sites can be selected, expanded and finally differentiated towards the desired MSC-subtype. The possibility and feasibility to obtain patient-specific, VW-MSCs from fibroblasts in large amounts by direct lineage conversion using VW-MSC-specific transcription factors will potentially open avenues towards novel, MSC-based therapies. The therapeutic function of MSCs is supposed to be mediated through a ‘hit/kiss and run’ mechanism [60, 93–95], thus indicating that continuous presence of the cells is not necessary. Instead, there is growing evidence MSCs may primarily act by a paracrine mechanism, namely by secreting exosomes/microvesicles. MSC-derived exosomes are smaller and less complex than their parental cells, and less immunogenic because of their lower content of membrane-bound proteins [96–100]. In line with these findings, we presented here that primary human VW-MSCs as well as iVW-MSCs expressed significantly higher amounts of exosome-associated membrane-proteins than fibroblasts. In agreement with their immunosuppressive activity, they expressed increased amounts of the immunosuppressive cytokine IDO. IDO is the first and rate-limiting enzyme involved in tryptophan catabolism, which suppresses immune responses by depleting local tryptophan concomitantly leading to the accumulation of tryptophan metabolites such as kynurenine, 3-hydroxyanthranillic acid, and quinolinic acid [101–103]. In addition, the major histocompatibility complex (MHC) class Ib molecule HLA-G, a well-known, MSC-secreted immunosuppressive molecule, was also expressed at increased levels by isolated human primary VW-MSCs and HOX-induced VW-MSCs generated from fibroblasts in vitro [104]. The cytokine secretion profile again revealed that generated iVW-MSCs were similar to ex vivo isolated hITA MSCs. These cytokine secretion profiles seemed to be common for MSCs, although the trophic nature of the MSCs that mainly depends on the cellular origin might account for different cytokine secretion levels [105]. MSC secretion profiles already revealed that classical pro-angiogenic cytokines, such as angiogenin, angiopoietins, VEGF, MCP1 and FGF2, can be detected in the conditioned medium of MSCs [106]. In line with these profiles, in disorders associated with insufficient angiogenesis MSCs turned out be a promising therapeutic approach [107, 108]. Herein, MSCs were supposed to establish a pro-angiogenic microenvironment by persistently secreting bioactive molecules that promote angiogenesis and microvascular network formation [109]. For example, MSC-derived angiogenin promoted primodial follicle survival and angiogenesis in transplanted human ovarian tissue [107]. However, the different in vitro and in vivo conditions might affect the composition of the profile of secreted molecules, which in turn might lead to differences in angiogenic capacities [110]. Thus, the data presented here may provide the molecular basis for further studies on MSC-assisted biological processes, such tissue homeostasis and regeneration as well as immune modulation.

### HOX-dependency of iVW-MSC-specific features

The context within a cell in which certain HOX genes are expressed is important, presumably due to the presence or absence of cofactors and/or coregulators, chromatin accessibility, and epigenetic changes accompanying cell identities [111]. For example, a timely induction of a *Hoxb1* transgene in embryonic stem cell (ESC)-derived neural stem cells resulted in the specification of these cells towards a hindbrain-specific identity and a concomitant repression of anterior neural identity [111, 112]. A doxycycline-inducible *Hoxb1* expression system was used here that initiated activation of an endogenous *Hoxb1* expression autoregulatory loop upon doxycycline addition. Accordingly, using an inducible system to express the *HOX* code in fibroblasts, we observed that HOX protein expression remained stable up to 21 days after doxycycline removal as well as the retaining clonogenicity and differentiation potential of directly converted fibroblast into iVW-MSCs. Besides anti-microbial and transgene-inducing functions, doxycycline was shown to improve the survival and proliferation of stem cells [113, 114]. These doxycycline effects were mediated by a direct modulation of different cellular pathways, e.g. activation of PI3 K/AKT intracellular signaling [113] or inhibition of matrix metalloproteinases (MMP) [115]. However, to achieve cell fate switching efficiently, lineage regulating genes must be uniformly induced [65]. Although HOX gene expression has been genetically manipulated, the identification of distinct HOX target genes in a given cell specification process is still urgently required. We could show that the introduced *HOX*-code in our iVW-MSC can be linked to the colony formation and differentiation potential as MSCs characteristics. In further studies, we will investigate whether this triple combination approach resembles the VW-MSC-specific master regulator for generating iVW-MSCs or whether a double combination or potentially single HOX candidates have the potential to be VW-MSC-specific key transcription factors, and which MSC characteristic(s) depend(s) on which introduced HOX gene. The identification of distinct HOX downstream-targets is also highly desired to gain novel insights into the molecular mechanism of MSC differentiation.

Conclusively, we report here a novel method for the direct conversion of human skin fibroblasts towards VW-MSCs using the VW-MSC-specific *HOX* code comprising the *HOX* genes *HOXB7*, *HOXC6* and *HOXC8* that directs cell fate conversion bypassing pluripotency. The resulting iVW-MSCs displayed classical multipotent MSC characteristics in vitro and selectively associated with vascular structures in vivo, thus likely representing true VW-MSCs. As important specific properties to discriminate MSCs from fibroblasts, iVW-MSCs behaved like primary ex vivo isolated VW-MSCs in terms of multipotency, clonogenicity and with respect to their therapeutic potential.

### Electronic supplementary material

Below is the link to the electronic supplementary material.

Supplementary material 1 (PDF 1800 kb)

Supplementary material 2 (XLSX 42 kb)

## Data Availability

The authors declare that all data supporting the findings of this study are available within the article and its supplementary information files or from the corresponding author upon reasonable request. The accession number for the comparative global profiling data reported in this article is ArrayExpress: E-MTAB-6743.

## References

[1] Rashidi A, DiPersio JF, Westervelt P, Abboud CN, Romee R (2016). Acute myeloid leukemia presenting with extensive bone marrow necrosis, leukemia cutis and testicular involvement: successful treatment with allogeneic hematopoietic stem cell transplantation. Bone Marrow Transplant.

[2] Faiella W, Atoui R (2016). Therapeutic use of stem cells for cardiovascular disease. Clin Transl Med.

[3] Siddiqi F, Wolfe JH (2016). Stem cell therapy for the central nervous system in lysosomal storage diseases. Hum Gene Ther.

[4] Squillaro T, Peluso G, Galderisi U (2016). Clinical trials with mesenchymal stem cells: an update. Cell Transplant.

[5] Sage EK, Thakrar RM, Janes SM (2016). Genetically modified mesenchymal stromal cells in cancer therapy. Cytotherapy.

[6] Glennie S, Soeiro I, Dyson PJ, Lam EW, Dazzi F (2005). Bone marrow mesenchymal stem cells induce division arrest anergy of activated T cells. Blood.

[7] Conese M, Carbone A, Castellani S, Di Gioia S (2013). Paracrine effects and heterogeneity of marrow-derived stem/progenitor cells: relevance for the treatment of respiratory diseases. Cells Tissues Organs.

[8] De Becker A, Riet IV (2016). Homing and migration of mesenchymal stromal cells: how to improve the efficacy of cell therapy?. World J Stem Cells.

[9] Leibacher J, Henschler R (2016). Biodistribution, migration and homing of systemically applied mesenchymal stem/stromal cells. Stem Cell Res Ther.

[10] Caplan AI, Dennis JE (2006). Mesenchymal stem cells as trophic mediators. J Cell Biochem.

[11] Devine SM, Cobbs C, Jennings M, Bartholomew A, Hoffman R (2003). Mesenchymal stem cells distribute to a wide range of tissues following systemic infusion into nonhuman primates. Blood.

[12] de Witte SFH, Luk F, Sierra Parraga JM, Gargesha M, Merino A, Korevaar SS, Shankar AS, O’Flynn L, Elliman SJ, Roy D, Betjes MGH, Newsome PN, Baan CC, Hoogduijn MJ (2018). Immunomodulation By therapeutic mesenchymal stromal cells (MSC) is triggered through phagocytosis of MSC By monocytic cells. Stem Cells.

[13] Lohan P, Treacy O, Griffin MD, Ritter T, Ryan AE (2017). Anti-donor immune responses elicited by allogeneic mesenchymal stem cells and their extracellular vesicles: are we still learning?. Front Immunol.

[14] Cho J, D’Antuono M, Glicksman M, Wang J, Jonklaas J (2018). A review of clinical trials: mesenchymal stem cell transplant therapy in type 1 and type 2 diabetes mellitus. Am J Stem Cells.

[15] Galipeau J, Sensebe L (2018). Mesenchymal stromal cells: clinical challenges and therapeutic opportunities. Cell Stem Cell.

[16] Turnbull MT, Zubair AC, Meschia JF, Freeman WD (2019). Mesenchymal stem cells for hemorrhagic stroke: status of preclinical and clinical research. NPJ Regenerative Med.

[17] Borakati A, Mafi R, Mafi P, Khan WS (2018). A systematic review and meta-analysis of clinical trials of mesenchymal stem cell therapy for cartilage repair. Curr Stem Cell Res Ther.

[18] Ward MR, Abadeh A, Connelly KA (2018). Concise review: rational use of mesenchymal stem cells in the treatment of ischemic heart disease. Stem Cells Transl Med.

[19] Connick P, Chandran S (2014). Mesenchymal stromal cell transplantation modulates neuroinflammatory milieu in amyotrophic lateral sclerosis. Cytotherapy.

[20] Connick P, Kolappan M, Crawley C, Webber DJ, Patani R, Michell AW, Du MQ, Luan SL, Altmann DR, Thompson AJ, Compston A, Scott MA, Miller DH, Chandran S (2012). Autologous mesenchymal stem cells for the treatment of secondary progressive multiple sclerosis: an open-label phase 2a proof-of-concept study. Lancet Neurol.

[21] Bader P, Kuci Z, Bakhtiar S, Basu O, Bug G, Dennis M, Greil J, Barta A, Kallay KM, Lang P, Lucchini G, Pol R, Schulz A, Sykora KW, von Luettichau I, Herter-Sprie G, Uddin MA, Jenkin P, Alsultan A, Buechner J, Stein J, Kelemen A, Jarisch A, Soerensen J, Salzmann-Manrique E, Hutter M, Schafer R, Seifried E, Klingebiel T, Bonig H, Kuci S (2018). Effective treatment of steroid and therapy-refractory acute graft-versus-host disease with a novel mesenchymal stromal cell product (MSC-FFM). Bone Marrow Transplant.

[22] Fujii S, Miura Y, Fujishiro A, Shindo T, Shimazu Y, Hirai H, Tahara H, Takaori-Kondo A, Ichinohe T, Maekawa T (2017). Graft-versus-host disease amelioration by human bone marrow mesenchymal stromal/stem cell-derived extracellular vesicles is associated with peripheral preservation of naive T cell populations. Stem Cells.

[23] Yu Y, Liu Y, Zong C, Yu Q, Yang X, Liang L, Ye F, Nong L, Jia Y, Lu Y, Han Z (2016). Mesenchymal stem cells with Sirt1 overexpression suppress breast tumor growth via chemokine-dependent natural killer cells recruitment. Sci Rep.

[24] Marofi F, Vahedi G, Biglari A, Esmaeilzadeh A, Athari SS (2017). Mesenchymal stromal/stem cells: a new era in the cell-based targeted gene therapy of cancer. Front Immunol.

[25] Le Blanc K, Davies LC (2018). MSCs-cells with many sides. Cytotherapy.

[26] Le Blanc K, Frassoni F, Ball L, Locatelli F, Roelofs H, Lewis I, Lanino E, Sundberg B, Bernardo ME, Remberger M, Dini G, Egeler RM, Bacigalupo A, Fibbe W, Ringden O, Developmental Committee of the European Group for B, Marrow T (2008). Mesenchymal stem cells for treatment of steroid-resistant, severe, acute graft-versus-host disease: a phase II study. Lancet.

[27] Jin HJ, Bae YK, Kim M, Kwon SJ, Jeon HB, Choi SJ, Kim SW, Yang YS, Oh W, Chang JW (2013). Comparative analysis of human mesenchymal stem cells from bone marrow, adipose tissue, and umbilical cord blood as sources of cell therapy. Int J Mol Sci.

[28] Kern S, Eichler H, Stoeve J, Kluter H, Bieback K (2006). Comparative analysis of mesenchymal stem cells from bone marrow, umbilical cord blood, or adipose tissue. Stem Cells.

[29] Zhu Y, Yang Y, Zhang Y, Hao G, Liu T, Wang L, Yang T, Wang Q, Zhang G, Wei J, Li Y (2014). Placental mesenchymal stem cells of fetal and maternal origins demonstrate different therapeutic potentials. Stem Cell Res Ther.

[30] Gotherstrom C, West A, Liden J, Uzunel M, Lahesmaa R, Le Blanc K (2005). Difference in gene expression between human fetal liver and adult bone marrow mesenchymal stem cells. Haematologica.

[31] Ergun S, Tilki D, Klein D (2011). Vascular wall as a reservoir for different types of stem and progenitor cells. Antioxid Redox Signal.

[32] Klein D, Weisshardt P, Kleff V, Jastrow H, Jakob HG, Ergun S (2011). Vascular wall-resident CD44+ multipotent stem cells give rise to pericytes and smooth muscle cells and contribute to new vessel maturation. PLoS One.

[33] Jung Y, Bauer G, Nolta JA (2012). Concise review: induced pluripotent stem cell-derived mesenchymal stem cells: progress toward safe clinical products. Stem Cells.

[34] Kimbrel EA, Kouris NA, Yavanian GJ, Chu J, Qin Y, Chan A, Singh RP, McCurdy D, Gordon L, Levinson RD, Lanza R (2014). Mesenchymal stem cell population derived from human pluripotent stem cells displays potent immunomodulatory and therapeutic properties. Stem Cells Dev.

[35] Ma T, Xie M, Laurent T, Ding S (2013). Progress in the reprogramming of somatic cells. Circ Res.

[36] Okano H, Nakamura M, Yoshida K, Okada Y, Tsuji O, Nori S, Ikeda E, Yamanaka S, Miura K (2013). Steps toward safe cell therapy using induced pluripotent stem cells. Circ Res.

[37] Klein D (2017). iPSCs-based generation of vascular cells: reprogramming approaches and applications.

[38] Lin L, Bolund L, Luo Y (2016). Towards personalized regenerative cell therapy: mesenchymal stem cells derived from human induced pluripotent stem cells. Curr Stem Cell Res Ther.

[39] Sabapathy V, Kumar S (2016). hiPSC-derived iMSCs: NextGen MSCs as an advanced therapeutically active cell resource for regenerative medicine. J Cell Mol Med.

[40] Frobel J, Hemeda H, Lenz M, Abagnale G, Joussen S, Denecke B, Saric T, Zenke M, Wagner W (2014). Epigenetic rejuvenation of mesenchymal stromal cells derived from induced pluripotent stem cells. Stem Cell Rep.

[41] Sheyn D, Ben-David S, Shapiro G, De Mel S, Bez M, Ornelas L, Sahabian A, Sareen D, Da X, Pelled G, Tawackoli W, Liu Z, Gazit D, Gazit Z (2016). Human iPSCs differentiate into functional MSCs and repair bone defects. Stem Cells Transl Med.

[42] Chen YS, Pelekanos RA, Ellis RL, Horne R, Wolvetang EJ, Fisk NM (2012). Small molecule mesengenic induction of human induced pluripotent stem cells to generate mesenchymal stem/stromal cells. Stem Cells Transl Med.

[43] Kurian L, Sancho-Martinez I, Nivet E, Aguirre A, Moon K, Pendaries C, Volle-Challier C, Bono F, Herbert JM, Pulecio J, Xia Y, Li M, Montserrat N, Ruiz S, Dubova I, Rodriguez C, Denli AM, Boscolo FS, Thiagarajan RD, Gage FH, Loring JF, Laurent LC, Izpisua Belmonte JC (2013). Conversion of human fibroblasts to angioblast-like progenitor cells. Nat Methods.

[44] Klein D (2018). iPSCs-based generation of vascular cells: reprogramming approaches and applications. Cell Mol Life Sci: CMLS.

[45] Steens J, Klein D (2018). Current strategies to generate human mesenchymal stem cells in vitro. Stem cells international.

[46] Chen F, Zhang G, Yu L, Feng Y, Li X, Zhang Z, Wang Y, Sun D, Pradhan S (2016). High-efficiency generation of induced pluripotent mesenchymal stem cells from human dermal fibroblasts using recombinant proteins. Stem Cell Res Ther.

[47] Lai PL, Lin H, Chen SF, Yang SC, Hung KH, Chang CF, Chang HY, Lu FL, Lee YH, Liu YC, Huang HC, Lu J (2017). Efficient generation of chemically induced mesenchymal stem cells from human dermal fibroblasts. Sci Rep.

[48] Steens J, Zuk M, Benchellal M, Bornemann L, Teichweyde N, Hess J, Unger K, Gorgens A, Klump H, Klein D (2017). In vitro generation of vascular wall-resident multipotent stem cells of mesenchymal nature from murine induced pluripotent stem cells. Stem Cell Rep.

[49] Klein D, Benchellal M, Kleff V, Jakob HG, Ergun S (2013). Hox genes are involved in vascular wall-resident multipotent stem cell differentiation into smooth muscle cells. Sci Rep.

[50] Stanurova J, Neureiter A, Hiber M, de Oliveira Kessler H, Stolp K, Goetzke R, Klein D, Bankfalvi A, Klump H, Steenpass L (2016). Angelman syndrome-derived neurons display late onset of paternal UBE3A silencing. Sci Rep.

[51] Klein D, Schmetter A, Imsak R, Wirsdorfer F, Unger K, Jastrow H, Stuschke M, Jendrossek V (2016). Therapy with multipotent mesenchymal stromal cells protects lungs from radiation-induced injury and reduces the risk of lung metastasis. Antioxid Redox Signal.

[52] Ritchie ME, Phipson B, Wu D, Hu Y, Law CW, Shi W, Smyth GK (2015). limma powers differential expression analyses for RNA-sequencing and microarray studies. Nucleic Acids Res.

[53] Benjamini Y, Hochberg Y (1995). Controlling the false discovery rate: a practical and powerful approach to multiple testing. J R Stat Soc Ser B (Methodol).

[54] Subramanian A, Kuehn H, Gould J, Tamayo P, Mesirov JP (2007). GSEA-P: a desktop application for gene set enrichment analysis. Bioinformatics.

[55] Subramanian A, Tamayo P, Mootha VK, Mukherjee S, Ebert BL, Gillette MA, Paulovich A, Pomeroy SL, Golub TR, Lander ES, Mesirov JP (2005). Gene set enrichment analysis: a knowledge-based approach for interpreting genome-wide expression profiles. Proc Natl Acad Sci USA.

[56] Zeilinger S, Kuhnel B, Klopp N, Baurecht H, Kleinschmidt A, Gieger C, Weidinger S, Lattka E, Adamski J, Peters A, Strauch K, Waldenberger M, Illig T (2013). Tobacco smoking leads to extensive genome-wide changes in DNA methylation. PLoS One.

[57] Muller F, Scherer M, Assenov Y, Lutsik P, Walter J, Lengauer T, Bock C (2019). RnBeads 2.0: comprehensive analysis of DNA methylation data. Genome Biol.

[58] Assenov Y, Muller F, Lutsik P, Walter J, Lengauer T, Bock C (2014). Comprehensive analysis of DNA methylation data with RnBeads. Nat Methods.

[59] Lee HY, An JH, Jung SE, Oh YN, Lee EY, Choi A, Yang WI, Shin KJ (2015). Genome-wide methylation profiling and a multiplex construction for the identification of body fluids using epigenetic markers. Forensic Sci Int Genet.

[60] Klein D, Steens J, Wiesemann A, Schulz F, Kaschani F, Rock K, Yamaguchi M, Wirsdorfer F, Kaiser M, Fischer JW, Stuschke M, Jendrossek V (2017). Mesenchymal stem cell therapy protects lungs from radiation-induced endothelial cell loss by restoring superoxide dismutase 1 expression. Antioxid Redox Signal.

[61] Wirsdorfer F, de Leve S, Cappuccini F, Eldh T, Meyer AV, Gau E, Thompson LF, Chen NY, Karmouty-Quintana H, Fischer U, Kasper M, Klein D, Ritchey JW, Blackburn MR, Westendorf AM, Stuschke M, Jendrossek V (2016). Extracellular adenosine production by ecto-5′-nucleotidase (CD73) enhances radiation-induced lung fibrosis. Can Res.

[62] Wiesemann A, Ketteler J, Slama A, Wirsdorfer F, Hager T, Rock K, Engel DR, Fischer JW, Aigner C, Jendrossek V, Klein D (2018). Inhibition of radiation-induced Ccl2 signaling protects lungs from vascular dysfunction and endothelial cell loss. Antioxid Redox Signal.

[63] Rohart F, Mason EA, Matigian N, Mosbergen R, Korn O, Chen T, Butcher S, Patel J, Atkinson K, Khosrotehrani K, Fisk NM, Le Cao KA, Wells CA (2016). A molecular classification of human mesenchymal stromal cells. PeerJ.

[64] Roson-Burgo B, Sanchez-Guijo F, Del Canizo C, De Las Rivas J (2016). Insights into the human mesenchymal stromal/stem cell identity through integrative transcriptomic profiling. BMC Genom.

[65] Bencsik R, Boto P, Szabo RN, Toth BM, Simo E, Balint BL, Szatmari I (2016). Improved transgene expression in doxycycline-inducible embryonic stem cells by repeated chemical selection or cell sorting. Stem cell research.

[66] Graf T, Enver T (2009). Forcing cells to change lineages. Nature.

[67] Hou PS, Chuang CY, Yeh CH, Chiang W, Liu HJ, Lin TN, Kuo HC (2017). Direct conversion of human fibroblasts into neural progenitors using transcription factors enriched in human ESC-derived neural progenitors. Stem Cell Rep.

[68] Nakamori D, Akamine H, Takayama K, Sakurai F, Mizuguchi H (2017). Direct conversion of human fibroblasts into hepatocyte-like cells by ATF5, PROX1, FOXA2, FOXA3, and HNF4A transduction. Sci Rep.

[69] Victor MB, Richner M, Olsen HE, Lee SW, Monteys AM, Ma C, Huh CJ, Zhang B, Davidson BL, Yang XW, Yoo AS (2018). Striatal neurons directly converted from Huntington’s disease patient fibroblasts recapitulate age-associated disease phenotypes. Nat Neurosci.

[70] Doppler SA, Deutsch MA, Lange R, Krane M (2015). Direct reprogramming-the future of cardiac regeneration?. Int J Mol Sci.

[71] Morris SA, Daley GQ (2013). A blueprint for engineering cell fate: current technologies to reprogram cell identity. Cell Res.

[72] Lee TI, Young RA (2013). Transcriptional regulation and its misregulation in disease. Cell.

[73] Chambers SM, Studer L (2011). Cell fate plug and play: direct reprogramming and induced pluripotency. Cell.

[74] Vierbuchen T, Wernig M (2011). Direct lineage conversions: unnatural but useful?. Nat Biotechnol.

[75] Pang ZP, Yang N, Vierbuchen T, Ostermeier A, Fuentes DR, Yang TQ, Citri A, Sebastiano V, Marro S, Sudhof TC, Wernig M (2011). Induction of human neuronal cells by defined transcription factors. Nature.

[76] Huang P, Zhang L, Gao Y, He Z, Yao D, Wu Z, Cen J, Chen X, Liu C, Hu Y, Lai D, Hu Z, Chen L, Zhang Y, Cheng X, Ma X, Pan G, Wang X, Hui L (2014). Direct reprogramming of human fibroblasts to functional and expandable hepatocytes. Cell Stem Cell.

[77] Yamamoto K, Kishida T, Sato Y, Nishioka K, Ejima A, Fujiwara H, Kubo T, Yamamoto T, Kanamura N, Mazda O (2015). Direct conversion of human fibroblasts into functional osteoblasts by defined factors. Proc Natl Acad Sci USA.

[78] Nam YJ, Song K, Luo X, Daniel E, Lambeth K, West K, Hill JA, DiMaio JM, Baker LA, Bassel-Duby R, Olson EN (2013). Reprogramming of human fibroblasts toward a cardiac fate. Proc Natl Acad Sci USA.

[79] Dominici M, Le Blanc K, Mueller I, Slaper-Cortenbach I, Marini F, Krause D, Deans R, Keating A, Prockop D, Horwitz E (2006). Minimal criteria for defining multipotent mesenchymal stromal cells. The International Society for Cellular Therapy position statement. Cytotherapy.

[80] Yusuf B, Gopurappilly R, Dadheech N, Gupta S, Bhonde R, Pal R (2013). Embryonic fibroblasts represent a connecting link between mesenchymal and embryonic stem cells. Dev Growth Differ.

[81] Alt E, Yan Y, Gehmert S, Song YH, Altman A, Vykoukal D, Bai X (2011). Fibroblasts share mesenchymal phenotypes with stem cells, but lack their differentiation and colony-forming potential. Biol Cell.

[82] Halfon S, Abramov N, Grinblat B, Ginis I (2011). Markers distinguishing mesenchymal stem cells from fibroblasts are downregulated with passaging. Stem Cells Dev.

[83] Lv FJ, Tuan RS, Cheung KM, Leung VY (2014). Concise review: the surface markers and identity of human mesenchymal stem cells. Stem Cells.

[84] Denu RA, Nemcek S, Bloom DD, Goodrich AD, Kim J, Mosher DF, Hematti P (2016). Fibroblasts and mesenchymal stromal/stem cells are phenotypically indistinguishable. Acta Haematol.

[85] Jaager K, Islam S, Zajac P, Linnarsson S, Neuman T (2012). RNA-seq analysis reveals different dynamics of differentiation of human dermis- and adipose-derived stromal stem cells. PLoS One.

[86] Goya RG, Lehmann M, Chiavellini P, Canatelli-Mallat M, Herenu CB, Brown OA (2018). Rejuvenation by cell reprogramming: a new horizon in gerontology. Stem Cell Res Ther.

[87] Mertens J, Paquola ACM, Ku M, Hatch E, Bohnke L, Ladjevardi S, McGrath S, Campbell B, Lee H, Herdy JR, Goncalves JT, Toda T, Kim Y, Winkler J, Yao J, Hetzer MW, Gage FH (2015). Directly reprogrammed human neurons retain aging-associated transcriptomic signatures and reveal age-related nucleocytoplasmic defects. Cell Stem Cell.

[88] Lapasset L, Milhavet O, Prieur A, Besnard E, Babled A, Ait-Hamou N, Leschik J, Pellestor F, Ramirez JM, De Vos J, Lehmann S, Lemaitre JM (2011). Rejuvenating senescent and centenarian human cells by reprogramming through the pluripotent state. Genes Dev.

[89] Mulvany MJ (1999). Vascular remodelling of resistance vessels: can we define this?. Cardiovasc Res.

[90] Renna NF, de Las Heras N, Miatello RM (2013). Pathophysiology of vascular remodeling in hypertension. Int J Hypertens.

[91] Korshunov VA, Schwartz SM, Berk BC (2007). Vascular remodeling: hemodynamic and biochemical mechanisms underlying Glagov’s phenomenon. Arterioscler Thromb Vasc Biol.

[92] Gibbons GH, Dzau VJ (1994). The emerging concept of vascular remodeling. N Engl J Med.

[93] Ankrum JA, Ong JF, Karp JM (2014). Mesenchymal stem cells: immune evasive, not immune privileged. Nat Biotechnol.

[94] Ghannam S, Bouffi C, Djouad F, Jorgensen C, Noel D (2010). Immunosuppression by mesenchymal stem cells: mechanisms and clinical applications. Stem Cell Res Ther.

[95] Klein D (2016). Vascular wall-resident multipotent stem cells of mesenchymal nature within the process of vascular remodeling: cellular basis, clinical relevance, and implications for stem cell therapy. Stem Cells Int.

[96] Lou G, Chen Z, Zheng M, Liu Y (2017). Mesenchymal stem cell-derived exosomes as a new therapeutic strategy for liver diseases. Exp Mol Med.

[97] Du W, Zhang K, Zhang S, Wang R, Nie Y, Tao H, Han Z, Liang L, Wang D, Liu J, Liu N, Kong D, Zhao Q, Li Z (2017). Enhanced proangiogenic potential of mesenchymal stem cell-derived exosomes stimulated by a nitric oxide releasing polymer. Biomaterials.

[98] Nong K, Wang W, Niu X, Hu B, Ma C, Bai Y, Wu B, Wang Y, Ai K (2016). Hepatoprotective effect of exosomes from human-induced pluripotent stem cell-derived mesenchymal stromal cells against hepatic ischemia-reperfusion injury in rats. Cytotherapy.

[99] Pashoutan Sarvar D, Shamsasenjan K, Akbarzadehlaleh P (2016). Mesenchymal stem cell-derived exosomes: new opportunity in cell-free therapy. Adv Pharm Bull.

[100] Hu GW, Li Q, Niu X, Hu B, Liu J, Zhou SM, Guo SC, Lang HL, Zhang CQ, Wang Y, Deng ZF (2015). Exosomes secreted by human-induced pluripotent stem cell-derived mesenchymal stem cells attenuate limb ischemia by promoting angiogenesis in mice. Stem Cell Res Ther.

[101] Ling W, Zhang J, Yuan Z, Ren G, Zhang L, Chen X, Rabson AB, Roberts AI, Wang Y, Shi Y (2014). Mesenchymal stem cells use IDO to regulate immunity in tumor microenvironment. Can Res.

[102] Platten M, Wick W, Van den Eynde BJ (2012). Tryptophan catabolism in cancer: beyond IDO and tryptophan depletion. Can Res.

[103] Phinney DG, Pittenger MF (2017). Concise review: MSC-Derived exosomes for cell-free therapy. Stem Cells.

[104] Rizzo R, Campioni D, Stignani M, Melchiorri L, Bagnara GP, Bonsi L, Alviano F, Lanzoni G, Moretti S, Cuneo A, Lanza F, Baricordi OR (2008). A functional role for soluble HLA-G antigens in immune modulation mediated by mesenchymal stromal cells. Cytotherapy.

[105] Park CW, Kim KS, Bae S, Son HK, Myung PK, Hong HJ, Kim H (2009). Cytokine secretion profiling of human mesenchymal stem cells by antibody array. Int J Stem Cells.

[106] Bronckaers A, Hilkens P, Martens W, Gervois P, Ratajczak J, Struys T, Lambrichts I (2014). Mesenchymal stem/stromal cells as a pharmacological and therapeutic approach to accelerate angiogenesis. Pharmacol Ther.

[107] Zhang Y, Xia X, Yan J, Yan L, Lu C, Zhu X, Wang T, Yin T, Li R, Chang HM, Qiao J (2017). Mesenchymal stem cell-derived angiogenin promotes primodial follicle survival and angiogenesis in transplanted human ovarian tissue. Reprod Biol Endocrinol.

[108] Sherman LS, Shaker M, Mariotti V, Rameshwar P (2017). Mesenchymal stromal/stem cells in drug therapy: new perspective. Cytotherapy.

[109] Caplan AI, Correa D (2011). The MSC: an injury drugstore. Cell Stem Cell.

[110] Hsieh JY, Wang HW, Chang SJ, Liao KH, Lee IH, Lin WS, Wu CH, Lin WY, Cheng SM (2013). Mesenchymal stem cells from human umbilical cord express preferentially secreted factors related to neuroprotection, neurogenesis, and angiogenesis. PLoS One.

[111] Gouti M, Gavalas A (2008). Hoxb1 controls cell fate specification and proliferative capacity of neural stem and progenitor cells. Stem Cells.

[112] Bhatlekar S, Fields JZ, Boman BM (2018). Role of HOX genes in stem cell differentiation and cancer. Stem Cells Int.

[113] Chang MY, Rhee YH, Yi SH, Lee SJ, Kim RK, Kim H, Park CH, Lee SH (2014). Doxycycline enhances survival and self-renewal of human pluripotent stem cells. Stem Cell Rep.

[114] Chang MY, Oh B, Rhee YH, Lee SH (2015). Doxycycline supplementation allows for the culture of human ESCs/iPSCs with media changes at 3-day intervals. Stem Cell Res.

[115] Lee HH, O’Malley MJ, Friel NA, Chu CR (2013). Effects of doxycycline on mesenchymal stem cell chondrogenesis and cartilage repair. Osteoarthr Cartil.

[116] Kaltz N, Ringe J, Holzwarth C, Charbord P, Niemeyer M, Jacobs VR, Peschel C, Haupl T, Oostendorp RA (2010). Novel markers of mesenchymal stem cells defined by genome-wide gene expression analysis of stromal cells from different sources. Exp Cell Res.

[117] Sivanathan KN, Rojas-Canales D, Grey ST, Gronthos S, Coates PT (2017). Transcriptome profiling of IL-17A preactivated mesenchymal stem cells: a comparative study to unmodified and IFN-gamma modified mesenchymal stem cells. Stem Cells Int.

[118] Kubo H, Shimizu M, Taya Y, Kawamoto T, Michida M, Kaneko E, Igarashi A, Nishimura M, Segoshi K, Shimazu Y, Tsuji K, Aoba T, Kato Y (2009). Identification of mesenchymal stem cell (MSC)-transcription factors by microarray and knockdown analyses, and signature molecule-marked MSC in bone marrow by immunohistochemistry. Genes Cells devoted Mol Cell Mech.

