# Supplementary material for: Direct conversion of human fibroblasts into therapeutically active vascular wall-typical mesenchymal stem cells
Source: Cell Mol Life Sci. 2019 Nov 11;77(17):3401–22. doi: 10.1007/s00018-019-03358-0 (PMC7426315; doi:10.1007/s00018-019-03358-0)
Supplement: Supplementary file 1 — Supplementary material 1 (PDF 1800 kb) [file 18_2019_3358_MOESM1_ESM.pdf]

Supplementary Information

Direct conversion of human fibroblasts into therapeutically active vascular wall-typical mesenchymal stem cells

Jennifer Steens[1], Kristian Unger[2], Lea Klar[1], Anika Neureiter[3], Karolin Wieber[1], Julia Hess[2], Heinz G. Jakob[4], Hannes Klump[3], Diana Klein[1]

[1]Institute for Cell Biology (Cancer Research), University Hospital Essen, University of Duisburg-Essen, Essen, Germany
[2]Research Unit Radiation Cytogenetics, Helmholtz Zentrum München, German Research Center for Environmental Health GmbH, Neuherberg, Germany
[3]Institute for Transfusion Medicine, University Hospital Essen, University of Duisburg-Essen, Essen, Germany
[4]Department of Thoracic and Cardiovascular Surgery, West-German Heart and Vascular Center Essen, University Duisburg-Essen, Germany

Supplementary Figures

Supplementary Figure S1

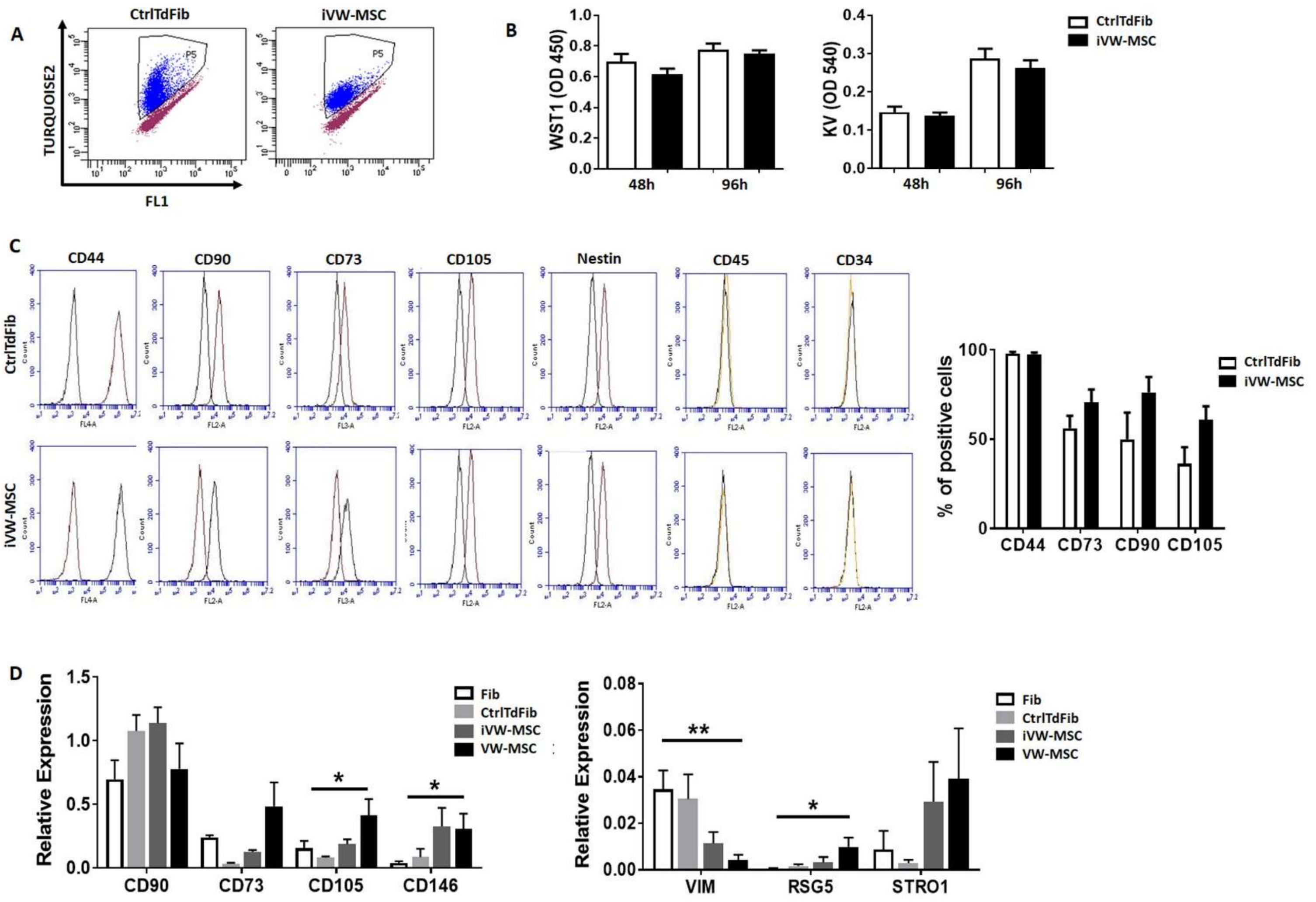

Supplementary Figure S2

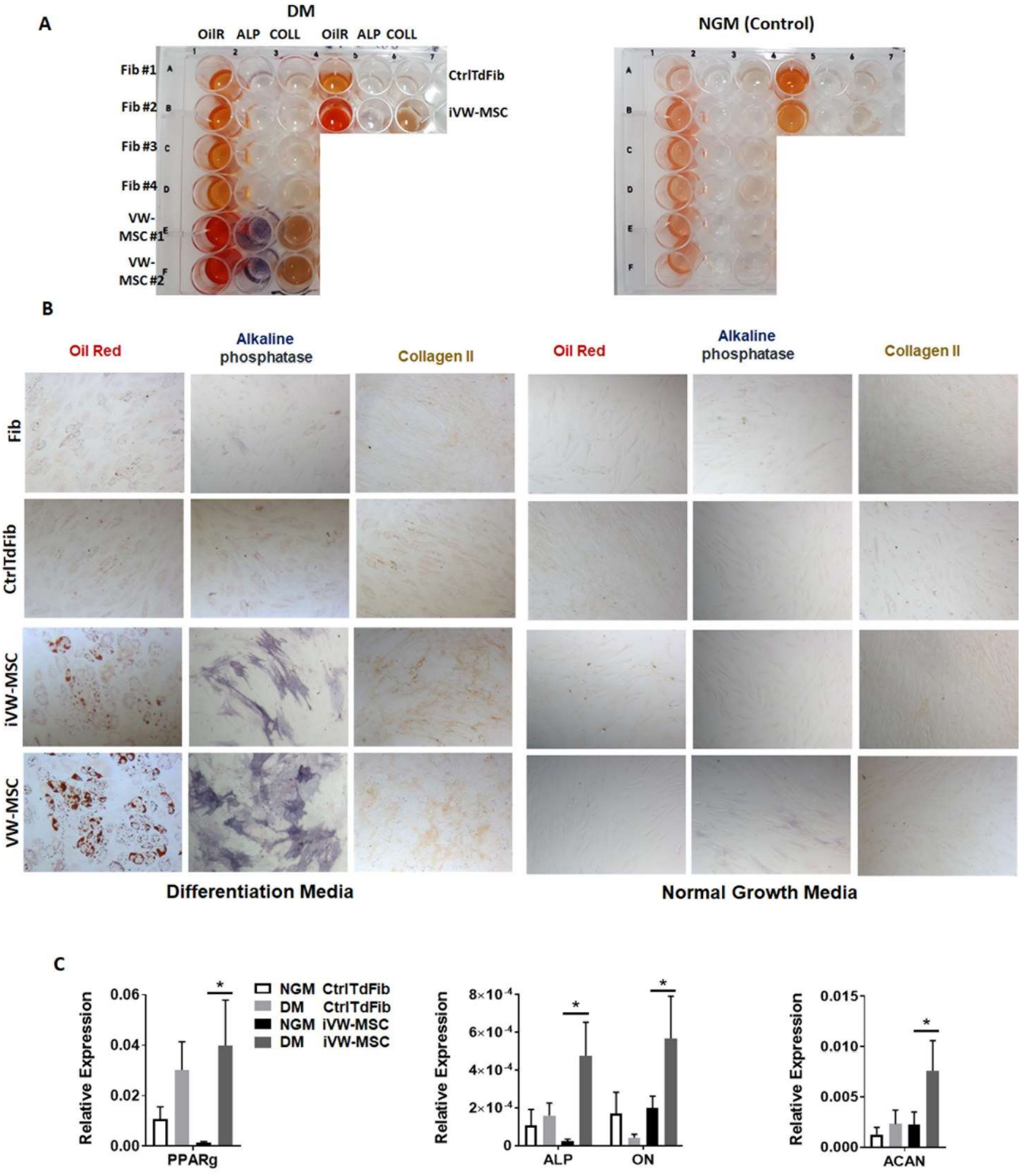

Supplementary Figure S3

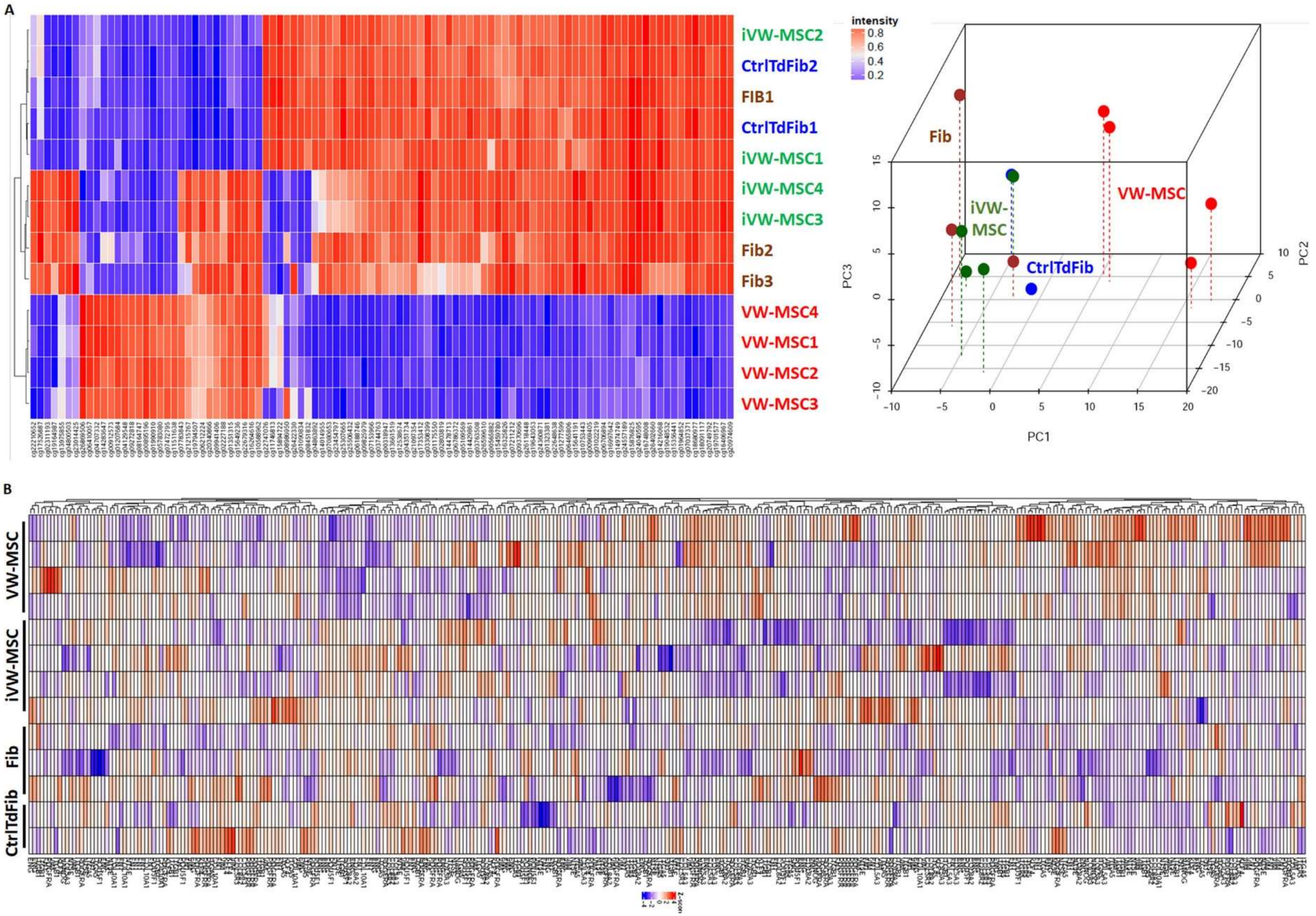

Supplementary Figure S4

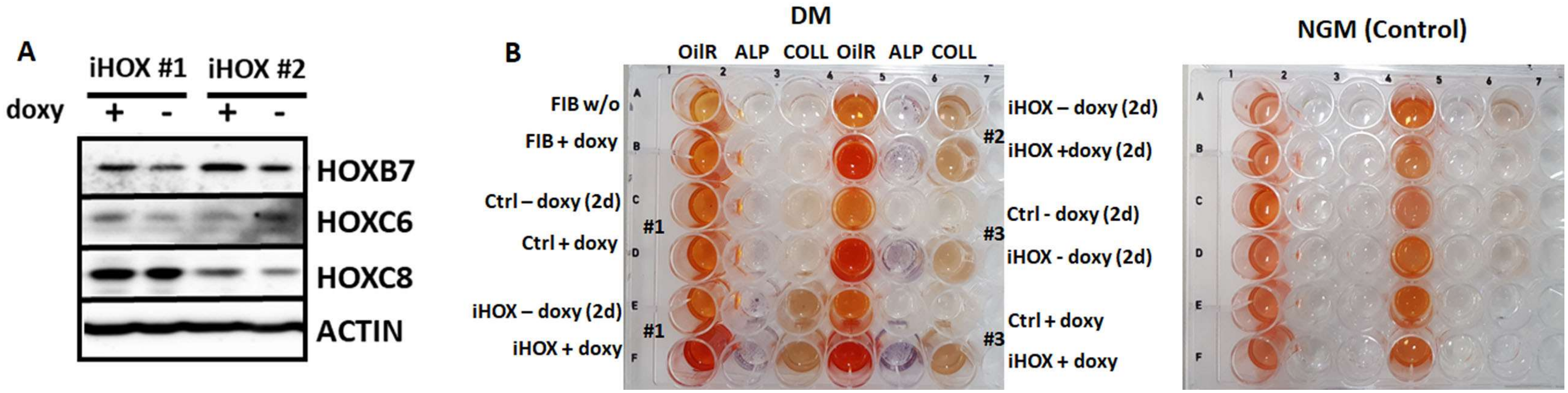

Supplementary Figure S5

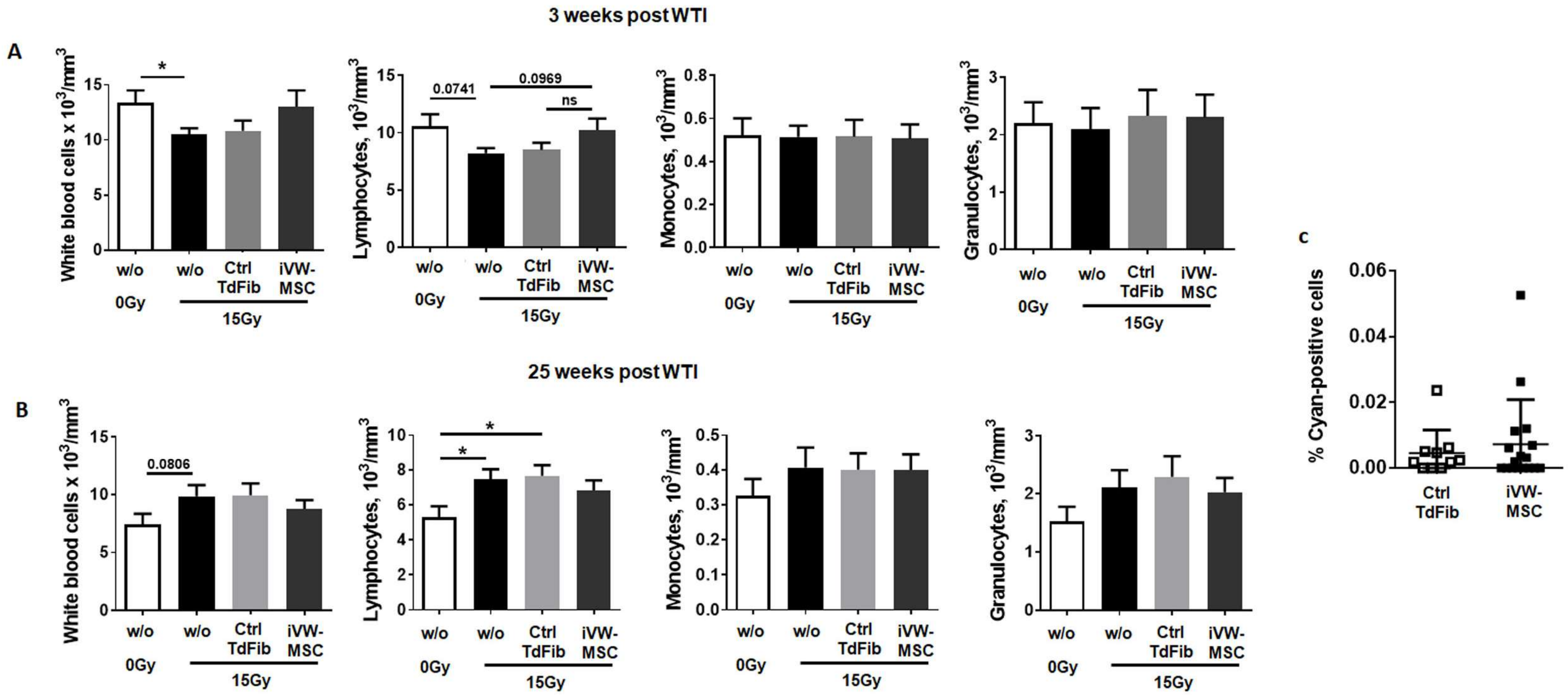

Supplementary Figure S6

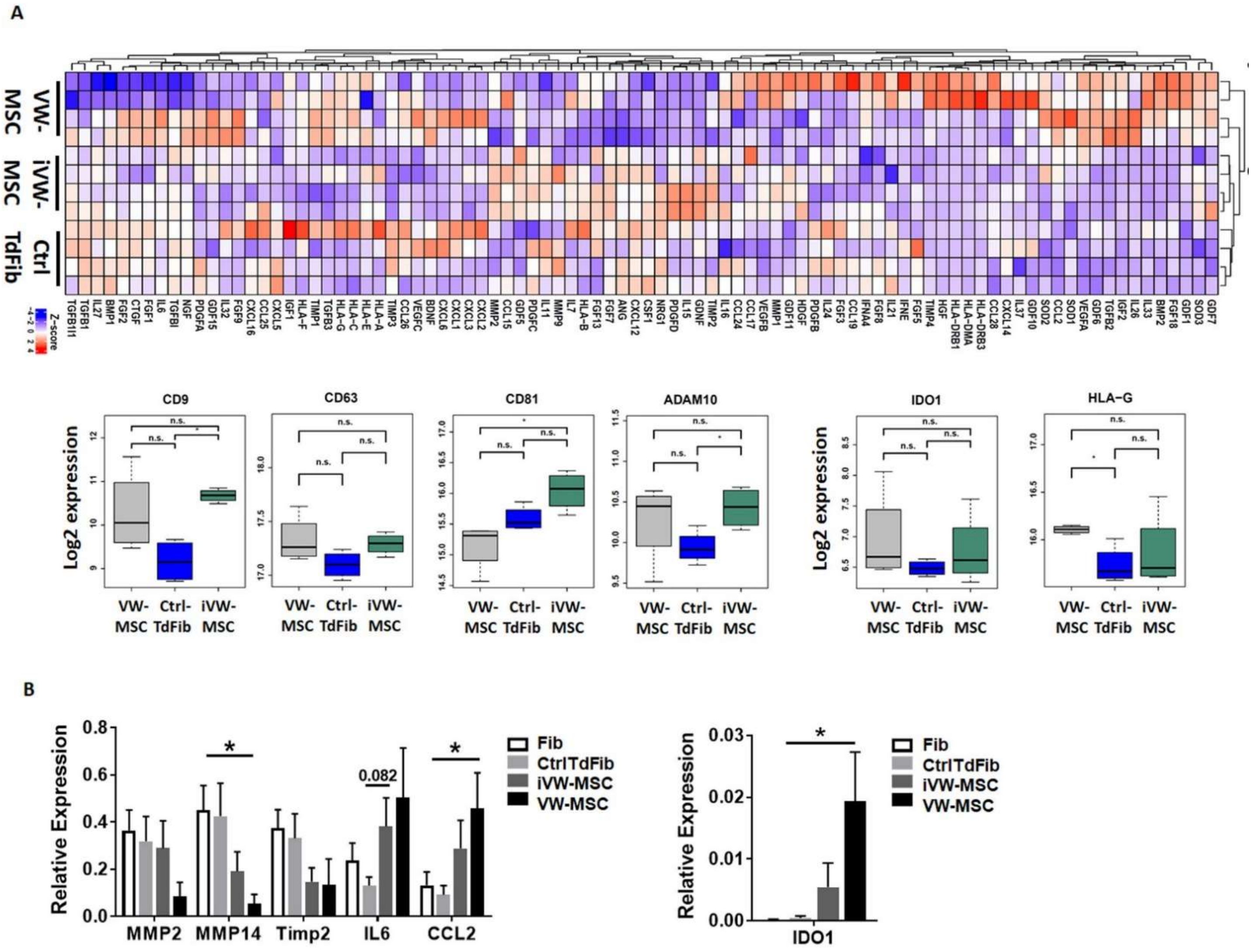

Supplementary Figure S7

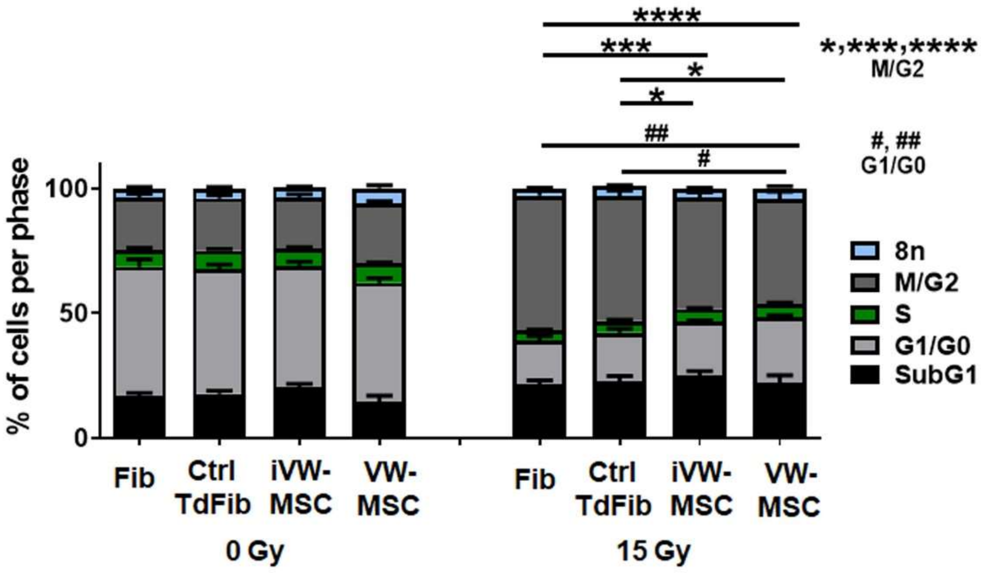

Supplemental Figure Legends

Supplementary Figure S1

Characterization of control-transduced fibroblasts and iVW-MSCs

(A) Representative scatter plots from the HOX- and control-vector (Ctrl) transduced fibroblasts only expressing *Turquoise2* are shown. The gated cell population (P5) was isolated by FACS. **(B)** Cell viability of cultured iVW-MSCs and control fibroblasts was assessed using a WST-1 reagent-based tetrazolium reduction assay. Proliferation was further analyzed with the crystal violet assay. Data are shown as means ± SEM of three independent experiments measured in quadruplets each. **(C)** Single cells suspension of control-transduced fibroblasts (CtrlTdFib) and iVW-MSCs were analyzed by FACS with indicated MSC marker antibodies (biological replicates: n=6-8 for each epitope and group). **(D)** Differential MSC marker gene expression levels in iVW-MSCs and control-transduced fibroblasts (CtrlTdFib) as compared to *ex vivo* isolated hITA (human internal thoracic artery)-derived VW-MSCs (positive control) and primary (non-transduced) fibroblasts (donor cells; negative control) were further confirmed by qRT-PCR. Relative amounts of transcripts of the indicated genes were determined by qRT-PCR (biological replicates: n=4-6 per group and gene; *P* by two-way ANOVA, followed by post-hoc Tukey's multiple comparisons test: $^{*}P \leq 0.05$; $^{**}P \leq 0.01$).

Supplementary Figure S2

Trilineage differentiation potential

The ability to differentiate into mesodermal linage cells was investigated for primary fibroblasts (FIB), control-transduced fibroblasts (CtrlTdFib), iVW-MSCs and hITA-derived VW-MSCs. Differentiation was observed within 14 days after induction of differentiation (DM) as shown by Oil red staining (adipocytes), by histochemical staining for alkaline phosphatase (ALP) of osteocytes, or by immunocytochemistry for collagen type II expression in chondrocytes. The trilineage differentiation potential was confirmed for iVW-MSCs and for hITA-derived VW-MSCs (positive control), while primary fibroblast as well as control-transduced fibroblast lack that differentiation potential. **(A)** Representative photographs of the cell culture plates are shown. (**B)** Representative photographs of the different conditions are shown. Magnification 400x (# indicates different donors). As control, respective cells were cultured in normal growth media (NGM). **(C)** Trilineage differentiation along the mesodermal lineage was further quantified after 14 days of culture within differentiation media by qRT-PCR analysis of peroxisome proliferator-activated receptor gamma (PPARg) for adipocytes, alkaline phosphatase (ALP) and osteocalcin (ON) for osteocytes, and aggrecan (ACAN) expression levels for chondrocytes. Data were presented as mean ± SEM (biological replicates: n=7 for each group and gene).

Supplementary Figure S3

DNA methylation analysis

(A) Hierarchical clustering of the beta-values (top 100 variants) of the global DNAm profiles of primary fibroblasts (FIB), control-transduced fibroblasts (CtrlTdFib), iVW-MSCs and hITA-

derived VW-MSCs are shown (biological replicates as indicated: VW-MSC n=4; HOX n=4, Ctrl n=2, FIB n=3). The principle component analysis of the global DNAm profiles is shown in the right diagram. **(B)** Hierarchical clustering of the beta-values of the global DNAm profiles from the classical MSC markers (Rohart MSC signature) of primary fibroblasts (FIB), control-transduced fibroblasts, iVW-MSCs (HOX) and hITA-derived VW-MSCs are shown (biological replicates as indicated: VW-MSC n=4; HOX n=4, Ctrl n=2, FIB n=3).

Supplementary Figure S4

Regulated expression of HOXB7, HOXC6 and HOXC8

(A) Primary human fibroblasts were transduced with the doxycycline inducible SIN vector encoding for the HOX code. Transduced cells (iHOX) were treated with doxycycline (0.2-0.5µg/ml) 48 hours after transduction, sorted for cyan fluorescence after expansion (additional 4-6 days) and then cultured in MSC medium. Western blot analysis of total HOXB7, HOXC6 and HOXC8 protein expression was performed from whole cell lysates of HOX-transduced and control fibroblasts with or without doxycycline withdrawal for 21 days. Beta-actin (ACTIN) was included as loading controls. # indicates fibroblasts derived from (2) different healthy donors. No changes of the doxycycline-induced HOXB7, HOXC6 and HOXC8 protein expression levels were detectable 21 days after doxycycline withdrawal. **(B)** Verification of doxycycline-induced conversion into MSCs. FACS-purified, iHOX-transduced and control doxycycline-treated fibroblasts were differentiated into adipocytes, osteocytes and chondrocytes at the indicated time points after doxycycline removal. Differentiation was observed within 14 days after induction of differentiation (DM) as shown by Oil red staining (dark red color) for adipocytes, by histochemical staining for alkaline phosphatase (ALP) visualizing osteocytes (dark blue color), or by immunocytochemistry for cartilage-specific collagen type II expressions of chondrocytes (dark brown color). Representative photographs of the cell culture plates are shown.

Supplementary Figure S5

Therapy with iVW-MSCs improved leukocytes blood levels of WTI mice

Total white blood cell counts as well as differential blood cell parameters (lymphocytes, monocytes, and granulocytes) were analyzed using peripheral blood samples (EDTA blood) within the inflammatory phase of WTI mice at 3 weeks (**A**; pneumonitic/acute phase) and 25 weeks (**B**; fibrotic/ chronic phase) after irradiation with or without therapeutically applied iVW-MSCs and control-transduced fibroblasts (CtrlTdFib) (n= 10-15 mice per group). **C** Peripheral blood samples were obtained 25 weeks after irradiation and circulating cyan-positive cells were quantified via flow cytometry. Data are presented as mean ± SEM (Ctrl: n=10 mice; HOX: n=17 mice). Data are presented as means ± SEM; $*P \leq 0.05$.

Supplementary Figure S6

Potential VW-MSCs-derived growth factors and cytokines accounting for a paracrine action

(A) Transcriptome heatmaps of growth factor and cytokine encoding genes extracted from the global gene expression profiles. Relative transcript amounts of exosome-associated surface markers and indicated immunomodulating genes are shown as boxplots (biological replicates: n=4 per group). **(B)** Differential candidate gene expression levels of known VW-MSC factors involved in tissue regeneration [1-3] in iVW-MSCs and control-transduced fibroblasts (CtrlTdFib) as compared to *ex vivo* isolated hITA (human internal thoracic artery)-derived VW-MSCs (positive control) and primary (non-transduced) fibroblasts (donor cells; negative control) were further confirmed by qRT-PCR. Relative amounts of transcripts of the indicated genes were determined by qRT-PCR (biological replicates: n=6-8 per group and gene; *P* by two-way ANOVA, followed by post-hoc Tukey's multiple comparisons test: $^{*}P \leq 0.05$).

Supplementary Figure S7

VW-MSCs-specific paracrine action affects the cell cycle of cultured endothelial cells

Cell cycle phases of endothelial cells treated with and without irradiation and subsequent conditioned medium (CM) treatment was analyzed 24 hours after treatment. Data are shown as means ±SEM of three independent experiments measured in duplicates each. $^{****,\#\#\#\#}P \leq 0.0001$, $^{***,\#\#\#}P \leq 0.001$, $^{**,\#\#}P \leq 0.01$ $^{*,\#}P \leq 0.05$, by two-way ANOVA followed by post-hoc Tukey’s test. Significances (each $P \leq 0.0001$) of the radiation-induced G1 reductions and the G/M arrests for each treatment condition were not indicated.

Supplementary Tables

Supplementary Table 1

Oligonucleotides used for qRT-PCR

Gene	Primer sequence (sense)	Primer sequence (anti-sense)
**HOXB7 3´UTR**	TTTGTGGACTGTGGGTCTGG	AGTGGTAGGTTTTGGGGCTC
**HOXB7 CDS**	GCCCTTTGAGCAGAACCTCT	AGTTCCTGAGCTTCGCATCC
**HOXB7_5´UTR**	TGCCTACAAATCATCCGGCC	TTTGTTCTGGGAAGGCTCCG
**HOXC6L_3´UTR**	GAAAGTCAGCTCTGGACCCC	GGGGAAAAGGGCCTGTAGAC
**HOXC6L_CDS**	CTGACCGAGCGACAGATCAA	CGCTTTTCCTCTTTTCCGCC
**HOXC6L_5´UTR**	TGTGGTTTGTCCGTTCCGAG	ACTGCTAATGAAGGGAGCGC
**HOXC8_3´UTR**	CTCTGTCTCACTCCTTGCCC	GGTGAGAGACAGACCGAGGA
**HOXC8 CDS**	ATTCTATGGCTACGAGGCGC	GCCTTGTCCTTCGCTACTGT
**HOXC8_5´UTR**	GAGGGGAGTTTCGGGGGTAC	GTACATGAAAACCCGCGGC
**wPRE**	CAGCTCCTTTCCGGGACTTT	CCATGGAAAGGACGTCAGCT
**CD90**	GGACTGAGATCCCAGAACCA	ACGAAGGCTCTGGTCCACTA
**CD73**	TAAAAGGTTCCACCCTGAAGAA	CTTCCTGTGGAAAACTTGATCC
**CD105**	ACTCCTCCCAAGGACACTTGTA	TGATGAGCTCGACAGGATATTG
**CD146**	CTGTGGAGTCGAGTGGTTTGTA	TAGAAAACAGGGACGGTGACTT
**STRO1**	GAAGCTAAAGTGGATTCAGGAGTA	AAGCAGGGGACCATTACA
**IDO1**	GCTGTTCCTTACTGCCAACTCT	CTTTACTGCAGTCTCCATCACG
**IL6**	GGTACATCCTCGACGGCATCT	AGTGCCTCTTTGCTGCTTTCAC
**PPARg**	GATCCAGTGGTTGCAGATTACA	CAAACCTGATGGCATTATGAGA
**Osteogenin**	TTCTGAGCCAGAAAGTGTGGTA	GCCCCTTTCTCTGTTTCTTTTT
**Aggrecan**	CTCCAATGACTCTGGGGTCTAC	CCAGGAAACTCATCCTTGTCTC

Specific primers were designed with the program Primer 3 (http://bioinfo.ut.ee/primer3-0.4.0/) based on available NCBI nucleotide CDS sequences and all primers used in our study were intron-spanning. PCR products are 200-300 bp in size.

Supplementary Table 2

Antibodies used in this study.

Antibody, Clone (host)	Supplier	Cat#	Dilution
Nestin, H85 (rabbit)	Santa Cruz Biotech	AP07829PU-N	1:200
Nestin (rabbit)	OriGene	AP07829PU-N	1:500
CFP (rabbit)	Sigma-Aldrich	SAB2108322	1.500

HoxB7, 747C4a (mouse)	Santa Cruz Biotech	sc-81292	1:200
HoxC6, B7 (mouse)	Santa Cruz Biotech	sc-376330	1:200
HoxC6 (rabbit)	Acris Antibodies	AP06660PU-N	1:200
HoxC8 (rabbit)	Bioss Inc.	bs-0394R	1:200
CD44, IM7 (rat)	Biolegend	103002	1:200
CD90, 3H1751 (mouse)	Novus Biologicals	NB-200-528	1:200
CD105, MEM-229 (mouse)	Acris Antibodies	AM03092PU-N	1:200
CD73, H300 (rabbit)	BD Bioscience	550741	1:100
CD73, AD2 (mouse)	BD Bioscience	550257	1:100
CD45, HI30 (mouse)	BD Bioscience	555483	1:100
CD34, QBEnd 10 (mouse)	DAKO	M7165	1:100
CD31, JC/70A (mouse)	DAKO	M0823	1:200
Actin, AC74 (mouse)	Sigma-Aldrich	A2228	1:5000

Supplementary Table 3

Epigenetic age prediction using the RnBeads software package tool MethylAger.

(See Supplementary Exel File.)

Supplementary Table 4

Gene sets used for GSEA.

(See Supplementary Exel File.)

Supplementary Experimental Procedures

Flow cytometry

For flow cytometric analysis, cells were treated with Trypsin/EDTA (Life Technologies) to generate single-cell suspensions, passed through a 40 µm cell strainer and counted. For each staining reaction, $1x10^5$ cells were incubated with 100 ng fluorochrome-coupled antibody (antigen-specific or isotype control) in a volume of 100-200 µl FACS buffer (5% (v/v) FBS in PBS, for 20 min at 4 °C. After addition of 1 ml FACS buffer and centrifugation (700 x g, 5 min), cells were resuspended in 200 µl FACS buffer and analyzed on a FACS Aria III (Becton Dickinson) using the FACSDiva software (Becton Dickinson). Kaluza® software (Beckman Coulter) was used for data analysis. Antibodies are listed in Table S2.

Supplementary References

1. Klein D, Steens J, Wiesemann A, Schulz F, Kaschani F, Rock K, Yamaguchi M, Wirsdorfer F, Kaiser M, Fischer JW, Stuschke M, Jendrossek V (2017) Mesenchymal Stem Cell Therapy Protects Lungs from Radiation-Induced Endothelial Cell Loss by Restoring Superoxide Dismutase 1 Expression. Antioxidants & redox signaling 26 (11):563-582. doi:10.1089/ars.2016.6748
2. Klein D, Schmetter A, Imsak R, Wirsdorfer F, Unger K, Jastrow H, Stuschke M, Jendrossek V (2016) Therapy with Multipotent Mesenchymal Stromal Cells Protects Lungs from Radiation-Induced Injury and Reduces the Risk of Lung Metastasis. Antioxidants & redox signaling 24 (2):53-69. doi:10.1089/ars.2014.6183
3. Wiesemann A, Ketteler J, Slama A, Wirsdorfer F, Hager T, Rock K, Engel DR, Fischer JW, Aigner C, Jendrossek V, Klein D (2019) Inhibition of Radiation-Induced Ccl2 Signaling Protects Lungs from Vascular Dysfunction and Endothelial Cell Loss. Antioxidants & redox signaling 30 (2):213-231. doi:10.1089/ars.2017.7458
